# Spherical Nucleic Acid Stabilized Cage Type Three‐Dimensional Electrochemiluminescence Probe for Sensitive and Amplification‐Free Diagnosis of Infectious Diseases

**DOI:** 10.1002/EXP.20240451

**Published:** 2026-03-14

**Authors:** Yu Fu, Wenlu Song, Jinyan Lin, Wei Wang, Lunjing Liu, Wenjie Wu, Huiyi Yang, Jingru Wang, Yang Yang, Bowen Shu, Yideng Jiang, Yuhui Liao

**Affiliations:** ^1^ NHC Key Laboratory of Metabolic Cardiovascular Diseases Research, Ningxia Key Laboratory of Vascular Injury and Repair Research Ningxia Medical University Yinchuan China; ^2^ Shenzhen Key Laboratory of Pathogen and Immunity, National Clinical Research Center for Infectious Disease, State Key Discipline of Infectious Disease Shenzhen Third People's Hospital Southern University of Science and Technology Shenzhen China; ^3^ Institute for Engineering Medicine Kunming Medical University Kunming China

**Keywords:** amplification‐free, electrochemiluminescence, metal‐organic framework, nucleic acid detection, point of care testing, spherical nucleic acid

## Abstract

Accurate nucleic acid‐based pathogen diagnosis is critical for clinical treatment and epidemic control. However, present gold‐standard testing methods based on nucleic acid amplification are limited by their time and labor‐intensive nature. Hence, we reported an amplification‐free assay that facilitates rapid, sensitive, and point‐of‐care detection of infectious diseases. Herein, spherical nucleic acid (SNA)‐stabilized cage‐type three‐dimensional electrochemiluminescence (ECL) reporter probe, constructed utilizing Ru(dcbpy)_3_Cl_2_‐doped NH_2_‐MIL‐101 (Fe) metal‐organic framework, was combined with a paper‐based bipolar electrode ECL sensor. Furthermore, surface modifications were performed using polydopamine and SNAs to further improve the stability. Notably, compared with the ordinary Ru(dcbpy)_3_Cl_2_, the modified ECL probe attained 10^3^‐fold enhancement in luminous efficiency. The developed ECL‐based nucleic acid biosensing system offers the advantages of simplicity, portability, low cost, and user‐friendly nucleic acid detection, with a sample‐to‐answer time of approximately 15 min. Additionally, the effectiveness of the developed assay in detecting pathogenic nucleic acid was assessed using Mpox and SARS‐Cov‐2, exhibiting a wide dynamic range from 33 to 10^10^ aM and limits of detection as low as 3 copies/µL. Furthermore, the assay exhibited 100% sensitivity and specificity when validated against quantitative polymerase chain reaction‐based detection using clinical samples. Altogether, the findings of this study show the efficacy of the novel developed SNA‐stabilized cage‐type 3D ECL probe‐enhanced nucleic acid assay, along with its potential as a promising paradigm for point‐of‐care diagnosis of infectious pathogens.

## Graphical abstract

This work developed a spherical nucleic acid stabilized cage‐type 3D electrochemiluminescence reporter probe for amplification‐free and ultrasensitive detection of pathogenic. Furthermore, a hand‐held detection device was designed, and we established a straightforward workflow enabling sample to answer within 15 min. This assay has excellent sensitivity and consistency in detecting pathogenic from various clinical samples.

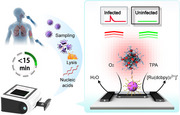

## 1 Introduction

Rapid and ultrasensitive point‐of‐care (POC) detection of pathogenic nucleic acids is crucial for infectious disease surveillance. In recent years, different outbreaks, such as the coronavirus disease 2019 (COVID‐19) pandemic and the reemergence of monkeypox (Mpox), have highlighted limitations of the centralized diagnostic laboratory model, including lengthy turnaround‐time [1, 2]. Despite the advantages of simple operation and rapidity of immunoassays, which make them an optimal POC test, their clinical utilities were limited to moderate sensitivity because of a long window period of several days to weeks, leading to missed detection and early spread. Reportedly, quantitative polymerase chain reaction (qPCR) remains the gold‐standard method for pathogen detection. However, it often presents limitations of time‐intensive (3–6 h) analytical procedures, dedicated infrastructure, and skilled personnel requirements, which largely limit its application outside of centralized laboratories [3, 4]. These limitations underscore the urgent need to develop novel POC diagnostic technologies for rapid and ultrasensitive detection of pathogenic nucleic acids.

Many studies have explored POC detection approaches for low‐level nucleic acids, which can be generally categorized into sequence replication [5, 6] and signal amplification [7, 8]. The sequence replication approaches, including loop‐mediated isothermal amplification [9, 10], recombinase polymerase amplification [11, 12, 13], rolling circle amplification (RCA) [14, 15, 16], strand displacement amplification [17], nucleic acid sequences based amplification [18, 19], nicking enzyme‐assisted amplification [20, 21], whole genome amplification [22, 23], helicase‐dependent amplification [24, 25], can replicate the target sequence to a detectable level without sophisticated analytical procedures and instruments compared with the requirements of thermal cycling‐based qPCR. However, despite merits such as simplicity, rapidity, and instrument‐free operation, these approaches are prone to non‐specific amplification, leading to false‐positive results and affecting the development of accurate and reliable POC testing [26]. In contrast, signal amplification approaches commonly detect the target via clustered regularly interspaced short palindromic repeats (CRISPR) [27], enzyme‐free amplification, field‐effect transistors (FETs) [28, 29], surface plasmon resonance (SPR) [30], and electrochemistry (EC) [31, 32]. For instance, the target‐dependent trans‐cleavage mechanism in CRISPR effector proteins (including CRISPR‐associated protein Cas12, Cas13, and Cas14 families) has been associated with highly specific target recognition and processive cleavage of nearby reporter molecules, providing an attractive strategy for nucleic acid detection. However, lacking target concentration and activity of the CRISPR/Cas system can affect the signal amplification efficiency, usually requiring the combination of isothermal amplification methods to obtain high sensitivity [33, 34]. Additionally, for the sensors of the signal amplification methods, fluorescence and colorimetry are conventional signal output methods with a low signal‐to‐noise ratio (*S*/*N*), which limits their sensitivity. Electrochemiluminescence (ECL) methods combine the advantages of chemiluminescence and EC, including a low background signal, high sensitivity, wide linear range, and easy integration of microelectronic systems. Furthermore, owing to these advantages, ECL methods have garnered extensive interest in application in developing amplification‐free and ultrasensitive POC nucleic acid detection.

Recently, various advances regarding amplification‐free and ultrasensitive ECL nucleic acid assays have been reported, including non‐labeling methods and enrichment of luminescent molecules to increase their amount, electron transfer, or luminous efficiency. For instance, the non‐labeling ECL nucleic acid assay has been reported with various ECL luminescent molecules, such as Ru complex ([Ru(phen)_2_dppz]^2+^) intercalating into double‐stranded DNA target (limit of detection [LOD] = 0.05 copies/µL) [35]. Notably, this method provided excellent sensitivity without the labeling process or PCR; however, this approach presented limitations of extensive assay time (7–8 h) for luminescent molecule intercalating and applicability for the single‐stranded DNA target. Labeling strategy for enriching luminescent molecules in carriers has been shown to provide considerable sensitivity in ECL nucleic acid assay without target replication [36]. For example, a dendritic polymer carrier‐based ECL probe enabling the chemical bonding of 8–16 [Ru(bpy)_3_]^2+^ molecules to a single carrier was reported, and it could carry out amplification‐free and sensitive (500 copies per reaction) analysis of Zika virus in a blood drop [37, 38]. Nevertheless, various factors, including the steric hindrance effect of the polymer carrier and the limited sites for grafting Ru(bpy)_3_^2+^ molecules, affected its further improvement in analytical performance. Consequently, a metal‐organic framework (MOF) was used as the carrier for loading more luminescent molecules and enhancing the electron transfer to address its limitations. Similarly, other studies have reported probes to improve ECL efficiency. For instance, in 2019, Wang et al. utilized MIL‐101(Al)‐NH_2_, presenting a high specific surface area and inherent mesoporous structure, to load Ru(bpy)_3_^2+^, fabricating MIL‐101(Al):Ru‐PEI‐Au complexes, which were then used for procalcitonin detection [39]. In 2021, Huang et al. developed a hollow hierarchical MOF (HH‐UiO‐66‐NH_2_), grafting Ru(bpy)_2_(mcpbpy)^2+^ onto the coordinatively unsaturated Zr_6_ nodes; this probe presented brilliant ECL emission [40]. In 2023, Yang et al. used a classic ECL luminophore, Ru(dcbpy)_3_^2+^, as the building block to fabricate a Ru‐complex‐based covalent‐MOF [41]. Such studies have shown how nanomaterials can greatly improve ECL efficiency, allowing for excellent assay sensitivity. However, these probes need to be assembled on the electrode before use, as in the liquid phase, leakage of the luminescent molecule due to unstable bonding or material degradation during chemical modification can substantially affect the overall performance.

Hence, this study aimed to develop a cage‐type three‐dimensional (3D) ECL probe‐based POC nucleic acid detection assay that allows for sensitive and amplification‐free detection of pathogenic nucleic acids. Additionally, the efficacy of the developed approach was assessed for SARS‐CoV‐2 and Mpox up to the single‐copy level. First, the cage‐type 3D ECL probe was synthesized to leverage the specific spatial structure of Fe‐MOF to increase the loading of luminescent molecule Ru(bpy)_3_Cl_2_ and enhance electron transfer. Meanwhile, polydopamine (PDA) and spherical nucleic acids (SNAs) were utilized to modify the probe surface to further enhance its stability and reduce non‐specific absorption. The surface‐modified Fe‐MOF reporter probe exhibited notably high efficiency in ECL and ultralow background signal. Furthermore, a hand‐held detection device was designed, and a straightforward workflow was established, enabling a sample‐to‐answer period of <15 min. This assay exhibits excellent sensitivity and consistency with the gold standard of reverse transcription qPCR (RT‐qPCR) in detecting SARS‐CoV‐2 and Mpox RNA from various clinical samples. Additionally, the programmable targeting capability of the probe makes it a promising approach for other pathogenic nucleic acid detection, with the requirement of tailoring the sequences of capture and reporter probes. Overall, the findings of this study show that the proposed assay has the potential to be a powerful diagnostic tool for the POC testing of infectious diseases.

## 2 Results and Discussion

### 2.1 Principle of the Cage‐Type 3D ECL Probe‐Enhanced Nucleic Acid Assay

**FIGURE 1:**
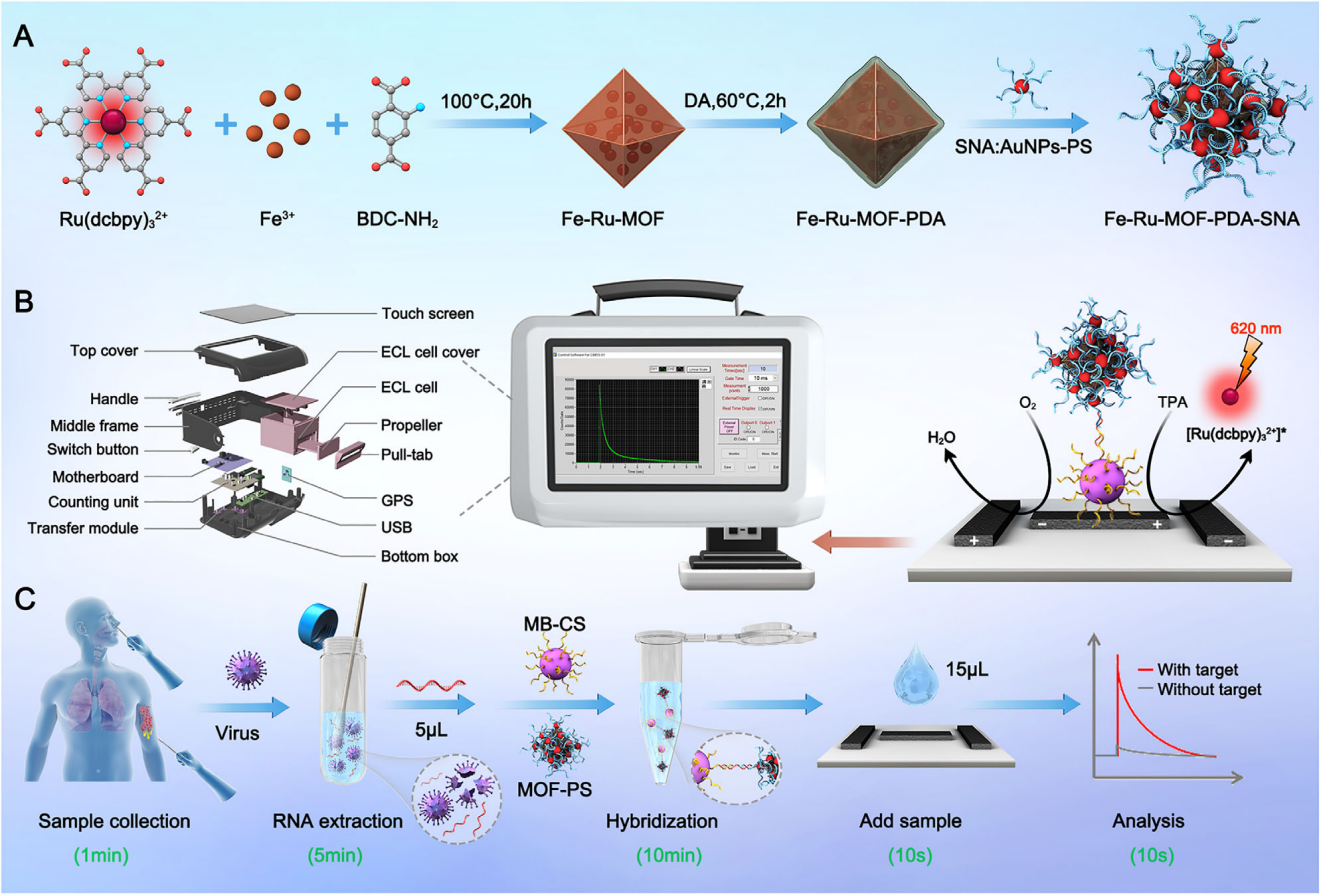
Schematic of the design of spherical nucleic acid (SNA)‐stabilized cage‐type three‐dimensional (3D) electrochemiluminescence (ECL) probe‐enhanced nucleic acid assay for infectious disease diagnosis. (A) Schematic illustration of the synthesis of the SNA‐stabilized cage‐type 3D ECL probe. (B) Structural diagram of the portable and easy‐to‐use device and paper‐based bipolar electrode ECL biosensor. (C) The complete sample‐to‐answer workflow of the ECL nucleic acid assay.

Herein, SNA‐stabilized cage‐type 3D ECL probe‐enhanced nucleic acid assay was proposed for rapid and accurate POC pathogenic nucleic acid detection. The probe was constructed by integrating ECL luminophores and Fe‐MOF as a cage‐type 3D carrier (Figure 1A). The stability of the probe was further improved by modifying its surface using the PDA and SNAs. To the best of our knowledge, this is the first time this modification has been reported. The ECL assay was simplified by creating a portable and easy‐to‐use device coupled with a paper‐based bipolar electrode (p‐BPE) ECL biosensor, allowing for POC testing. Overall, this device mainly consisted of a light‐proof optical cell, a p‐BPE device, a photomultiplier tube (PMT), a signal triggering and processing module, and a touch screen for human–machine interaction (Figure 1B). The p‐BPE device, which was easy to fabricate (Figures S1 and S2, Supporting Information) and low‐cost ($0.4) for single use‐highly desirable for POC test. It induced electrochemical reactions at its extremities upon the application of a sufficient voltage bias to the opposing driving electrodes without a counter electrode. The complete workflow included thermal lysis‐mediated sample preparation, RNA target enrichment by capture probe‐modified magnetic beads (MB‐CP), sandwich hybridization with cage‐type 3D ECL reporter probe, and addition of the hybridization complex and co‐reactant tripropylamine (TPA) on the p‐BPE device, which achieved a sample‐to‐answer time of approximately 15 min (Figure 1C). First, the collected body fluid samples (throat swab or pustule swab) were thermally lysed, and the target RNA was bound by MB‐CP via base stacking interaction. Next, the bound RNA targets were isolated from the lysate using a magnet, which facilitated target enrichment for improving assay sensitivity. The sandwiched complexes were further purified through magnetic separation, and unbound reporter probes were removed. Afterward, the purified sandwich complexes, together with co‐reagent TPA, were dropped on the p‐BPE. Under the 20 V potential, Ru^2+^ (doped in Fe‐MOF) and TPA lost an electron each, changing to Ru^3+^ and TPA^•+^, respectively. Subsequently, TPA^•+^ deprotonated to generate TPA^•^, which further reacted with Ru^3+^ to form the excited state Ru^2+*^. Finally, Ru^2+*^ was relaxed to the ground state, yielding superior ECL emission. The entire workflow of the amplification‐free and ultrasensitive assay was realized without the need for dedicated infrastructure and a sophisticated instrument.

### 2.2 Construction of the SNA‐Stabilized Cage‐Type 3D ECL Probe

**FIGURE 2:**
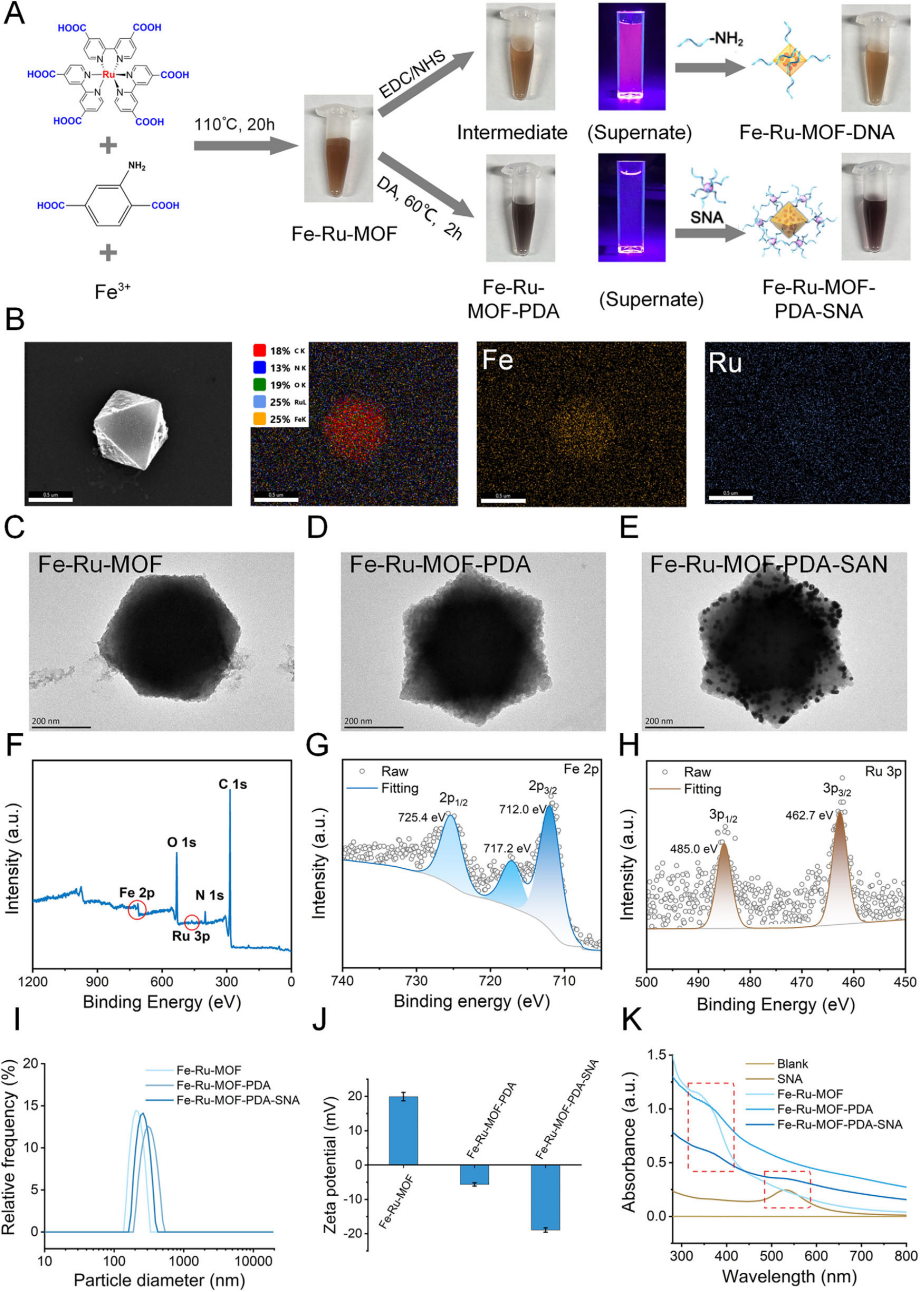
Construction and characterization of the spherical nucleic acid (SNA) cage‐type three‐dimensional (3D) electrochemiluminescence (ECL) reporter probe prepared using Fe‐metal‐organic framework (MOF). (A) Schematic showing the synthesis of Fe‐Ru‐MOF‐PDA‐SNA. (B) Scanning electron microscopy and mapping images of C, N, O, Fe, and Ru in Fe‐Ru‐MOF. (C–E) Transmission electron microscopy image of (C) Fe‐Ru‐MOF, (D) Fe‐Ru‐MOF‐PDA, and (E) Fe‐Ru‐MOF‐PDA‐SNA. (F–H) X‐ray photoelectron spectra of (F) Survey, (G) Fe 2p, and (H) Ru 3P. (I) Dynamic light scattering diameters of Fe‐Ru‐MOF, Fe‐Ru‐MOF‐PDA, and Fe‐Ru‐MOF‐PDA‐SNA. (J) Zeta potentials of Fe‐Ru‐MOF, Fe‐Ru‐MOF‐PDA, and Fe‐Ru‐MOF‐PDA‐SNA. (K) Ultraviolet‐visible spectra of Fe‐Ru‐MOF, Fe‐Ru‐MOF‐PDA, Fe‐Ru‐MOF‐PDA‐SNA, and SNA.

Previously, we developed a dendritic Ru(bpy)_3_^2+^‐polymer‐based ECL reporter that achieved an LOD of 500 copies per reaction. Various carriers with a higher loading of efficient ECL luminescent molecule (Ru(bpy)_3_^2+^) were assessed to further enhance its performance. Because Fe‐MOF exhibits excellent water stability [42, 43], it was used as the carrier of luminescent molecules in this study. In the preparation of [Ru(dcbpy)_3_Cl_2_]‐doped Fe‐MOF (Fe‐Ru‐MOF), Fe^3+^ acted as a Lewis acid, and 2‐aminoterephthalic acid and Ru(dcbpy)_3_Cl_2_ acted as Lewis bases to form the MOF structure based on coordination between Fe^3+^ and carboxyl groups (Figure 2A). The morphology and elemental analysis showed that slight Ru(dcbpy)_3_Cl_2_ was successfully doped into the Fe‐MOF crystals (Figure 2B,C and Figure S3, Supporting Information), consistent with the findings of the X‐ray photoelectron spectroscopy (Figure 2F–H) and Fourier‐transform infrared spectroscopy (Figure S4, Supporting Information).

For the application of the probe in ultrasensitive detection of pathogenic nucleic acids, its stable modification is crucial to achieve high sensitivity with ultralow background. Conventionally, 1‐ethyl‐3‐(3‐dimethylaminopropyl) carbodiimide/N‐hydroxysuccinimide is used to activate carboxyl groups to connect probes with amino groups for MOF modification. However, activation of carboxyl groups affects coordination between Fe^3+^ and carboxyl groups, leading to MOF degradation and Ru(dcbpy)_3_Cl_2_ leakage (Figure 2A and Figure S5, Supporting Information). To address this problem, in this study, the surface of Fe‐Ru‐MOF was modified by in situ synthesis of the PDA layer by autoxidation of dopamine (DA) to generate modification sites for nucleic acid probe via hydrogen bonding and π–π stacking. SNA‐labeled reporter sequences were used as stabilizers to further improve probe‐loading and structure stability via the strong intermolecular forces between the SNAs and PDA. TEM results showed that both PDA and SNA were successfully and uniformly bound to the MOF surface without affecting the MOF morphology (Figure 2D,E). The results of dynamic light scattering diameters, zeta potential, and ultraviolet–visible spectra indicated the successful synthesis of the cage‐type 3D ECL probe (Figure 2I–K). Additionally, the non‐specific adsorption was assessed using DNA sequences homologous to the capture probe to block non‐specific sites on the Fe‐Ru‐MOF‐PDA‐SNA. The DNA sequence concentration was 100 µM, and the volume ranged from 0 to 20 µL. Following centrifugation (5000 rpm), the sediment was washed thrice with ultrapure water. Next, another DNA probe with a random sequence (concentration, 10 µM; volume, 2 µL), modified with fluorescein amidite (FAM), was incubated with the Fe‐Ru‐MOF‐PDA‐SNA at 37°C for 30 min. After centrifugation, the supernatant was collected and analyzed using a fluorescence spectrophotometer. Notably, as the volume of DNA sequence for blocking non‐specific sites increased to 10 µL, the fluorescence signal of the supernatant enhanced to the maximum value (Figure S6, Supporting Information), which was similar to the findings of 2 nM free DNA‐FAM probe. Overall, these results showed that the non‐specific adsorption can be avoided under the optimal experimental conditions.

### 2.3 Evaluation of the ECL Performance

**FIGURE 3:**
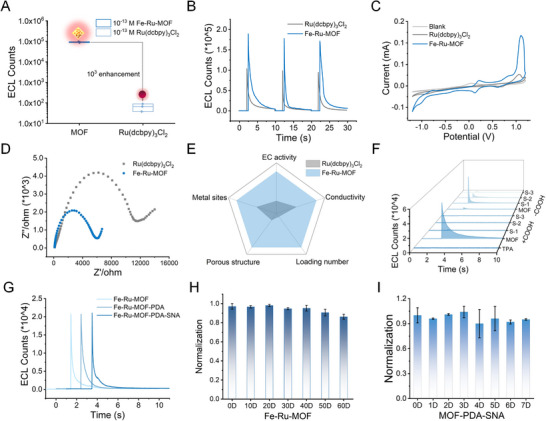
Evaluation of the electrochemiluminescence (ECL) performance of the ECL probe. (A) ECL profiles of Fe‐Ru‐MOF and Ru(dcbpy)_3_Cl_2_ at the same concentrations (10^−13^ M). (B) ECL profiles of Ru(dcbpy)_3_Cl_2_ and Fe‐Ru‐MOF at the same concentration of Ru(dcbpy)_3_Cl_2_ (0.1 µM). (C) Cyclic voltammetry. (D) Electrochemical impedance spectroscopy profiles of Ru(dcbpy)_3_Cl_2_ and Fe‐Ru‐MOF at the same concentration of Ru(dcbpy)_3_Cl_2_ (0.1 µM). (E) The performance of Fe‐Ru‐MOF and Ru(dcbpy)_3_Cl_2_. (F) ECL profiles of Ru(dcbpy)_3_Cl_2_ (with COOH) and Ru(bpy)_3_Cl_2_ (without COOH). (G) ECL profiles of the Fe‐Ru‐MOF, Fe‐Ru‐MOF‐PDA, and Fe‐Ru‐MOF‐PDA‐SNA (0.2 pM). (H) Normalized ECL signals of the Fe‐Ru‐MOF after soaking in deionized water for 0–60 days. (I) Normalized ECL signals of the MOF‐PDA‐SNA after soaking in deionized water for 7 days. Error bars represent s.d. (*n* > 3 replicates).

The ECL performance of the cage‐type 3D ECL probe was evaluated by comparing the luminescence efficiency of the Ru(dcbpy)_3_Cl_2_‐doped MOF crystals and the ordinary Ru(dcbpy)_3_Cl_2_ molecules at the same molar concentration (0.1 pM). Interestingly, Fe‐Ru‐MOF exhibited approximately 10^3^‐fold enhancement in ECL efficiency and 1.8 times stronger ECL intensity compared with that of the ordinary Ru(dcbpy)_3_Cl_2_ molecule at an equal concentration (Figure 3A,B). Consequently, the contribution of the unique structure of Fe‐MOF to its electrochemical efficiency was explored by examining its electrochemical properties through cyclic voltammetry and electrochemical impedance spectroscopy tests. Notably, both Ru(dcbpy)_3_Cl_2_ and Fe‐Ru‐MOF showed oxidation peaks at 1.1 V, but the current of Fe‐Ru‐MOF was significantly higher than that of Ru(dcbpy)_3_Cl_2_ (Figure 3C). Accordingly, results showed that the metal sites (Fe^3+^) in the MOF structure enhanced the conductivity of the MOFs (Figure 3D,E). Each Fe‐Ru‐MOF carrier presented a diameter of approximately 400 nm, with a load capacity for approximately 4.18 × 10^5^ Ru(dcbpy)_3_Cl_2_ molecules (section calculation formula for Fe‐Ru‐MOF is present in Supporting Information). This superior performance was attributed to the high loading of luminous molecules and the accelerated electron and ion transfer in the Fe‐Ru‐MOF structure. Overall, the results showed that the ECL efficiency of MOF‐Ru(dcbpy)_3_Cl_2_ was markedly higher than that of MOF‐Ru(bpy)_3_Cl_2_, with almost no ECL signal detected in its supernatant (Figure 3F).

Moreover, the results indicated that ECL luminescent molecules in Ru(dcbpy)_3_Cl_2_‐doped Fe‐MOF (MOF‐Ru(dcbpy)_3_Cl_2_) were more stable than the carboxyl group with Ru(bpy)_3_Cl_2_‐doped Fe‐MOF (MOF‐Ru(bpy)_3_Cl_2_). Additionally, PDA and SNA modifications did not affect the ECL performance of the MOF (Figure 3G). The results of soaking experiments revealed that the MOF and MOF‐based probes exhibited good chemical stability (Figure 3H,I). Altogether, a modified Fe‐Ru‐MOF probe was successfully fabricated, exhibiting superior ECL performance and excellent chemical stability, and thus, showing potential in amplification‐free and ultrasensitive detection.

### 2.4 Instrumentation of Portable ECL‐Based Nucleic Acid Biosensing System

**FIGURE 4:**
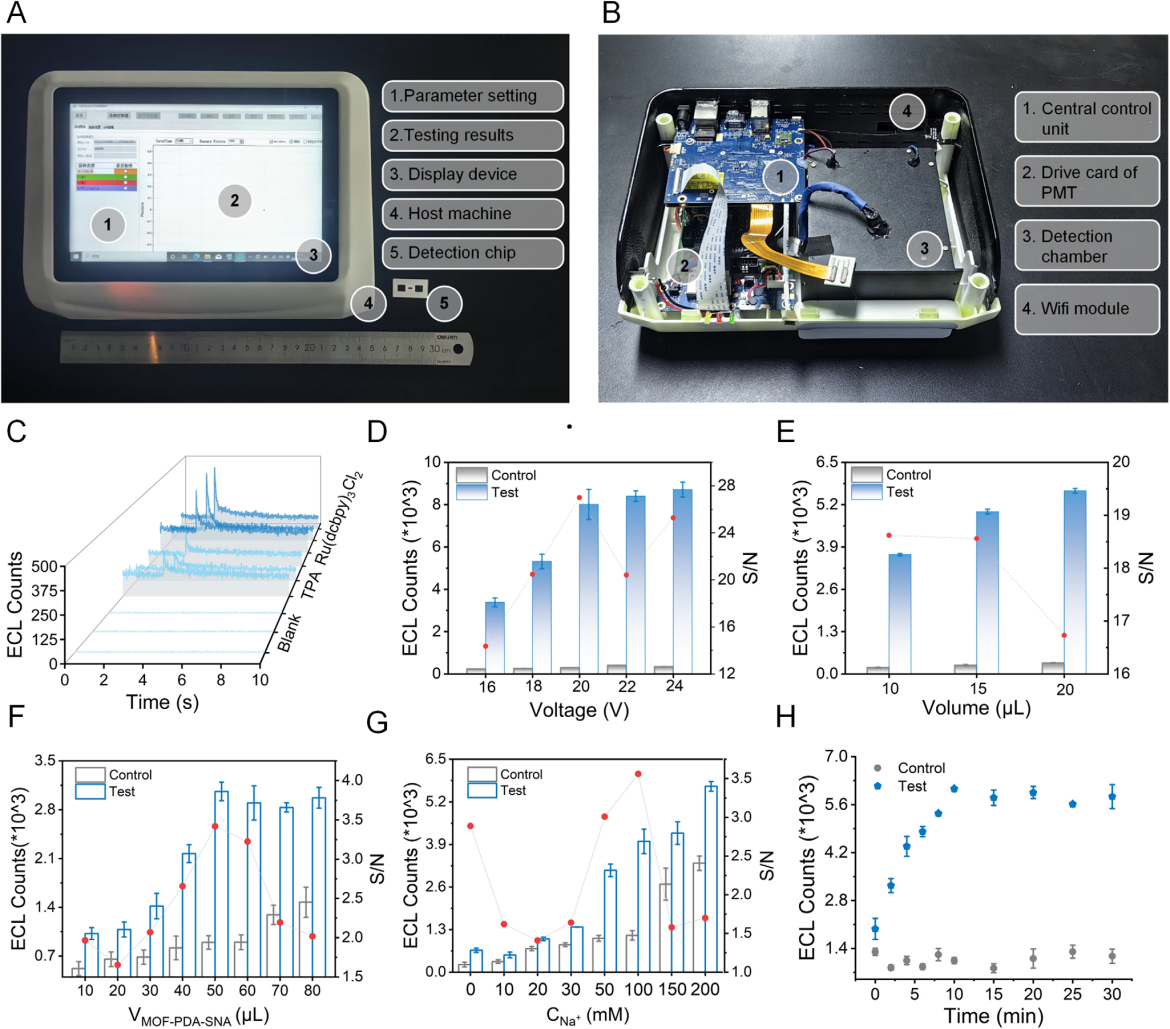
Overview of the portable electrochemiluminescence (ECL) nucleic acid biosensing system. (A) The photograph of an assembled portable biosensing system with a compact size. (B) The photograph of the system displays the main components. (C) ECL profiles of biosensing system under different conditions; blank: background signal of light‐proof optical cell; TPA: background signal of TPA; and Ru(dcbpy)_3_Cl_2_: ECL response of 10^5^ Ru(dcbpy)_3_Cl_2_. (D) Evaluation of the effect of different voltages of the biosensing system on the ECL sensor. (E) Evaluation of the effect of different volumes of test sample on the ECL sensor. (F) Evaluation of the effect of different volumes of MOF‐PDA‐SNA on the ECL sensor. (G) Evaluation of the effect of different concentrations of Na^+^ on the ECL sensor. (H) Time‐dependent ECL responses in the presence and absence of target.

The portable and easy‐to‐use ECL device (Figure 4A,B) primarily included a central control unit, driver card of PMT, detection chamber, PMT, and a touch screen (compact size of 30 × 20 × 15 cm^3^). The direct current transformer module was used to provide different driving voltages (0–20 V) for the ECL reaction on the p‐BPE. The vertical placement of the PMT above the p‐BPE and sealing inside the detection chamber maximized the reception of photons and minimized the influence of external photons, respectively. After the photoelectrons entered the PMT, they continuously collided with the lattice under the action of the electric field, producing more electron–hole pairs and multiplying the photocurrent, thereby significantly improving the sensitivity of the detector. The multiplied electrons were eventually collected by the anode, resulting in a measurable current signal. Subsequently, this current signal was amplified and converted into electrical signals by the driver card for data analysis in the central control unit. Finally, the detection results were output on the touch screen, which also served as the interface to change the detection conditions, such as voltage, measurement time, and gate time. To evaluate the sensitivity of the developed biosensing system (Figure 4C), the background of the light‐proof optical cell was investigated. Notably, less than 15 photon counts were detected in the blank. Further evaluation revealed that the ECL signal generated by 10^5^ Ru(dcbpy)_3_Cl_2_ was approximately twice that of the control (TPA), indicating the requirement of 10^5^ (Ru(dcbpy)_3_Cl_2_) molecules for effective ECL signal detection. Additionally, the major parameters that might affect the biosensing system performance, including the excitation voltage and loading sample volume, were investigated (Figure 4D,E). The excitation voltage at 20 V and 15 µL of the loading sample resulted in a higher signal‐to‐noise (*S*/*N*), and these parameters were applied in the following assays. To achieve optimal performance, the major parameters of the ECL assay were investigated, including the volume of the ECL probe and Na^+^ concentration (Figure 4F,G). Notably, 50 µL of ECL probe and 100 mM Na^+^ resulted in a higher *S*/*N*. This observation was attributed to the shielding effect of Na^+^ on charge exclusion between the ECL probe, MB‐CP, and targets, promoting the formation of a sandwich hybridization assay. Next, the required time for such target‐triggered sandwich hybridization assay was determined by monitoring the ECL counts at different time points (Figure 4H). Herein, an incubation time of 10 min was associated with the generation of the maximum ECL signal while maintaining a relatively low background. Collectively, these results show the simplicity of the proposed ECL assay, which allows for a rapid, low‐cost, user‐friendly, and sample‐to‐answer nucleic acid detection to address the urgent need for POC diagnosis of infectious diseases.

### 2.5 Performance Index of the Cage‐Type 3D ECL Probe‐Enhanced Nucleic Acid Assay

**FIGURE 5:**
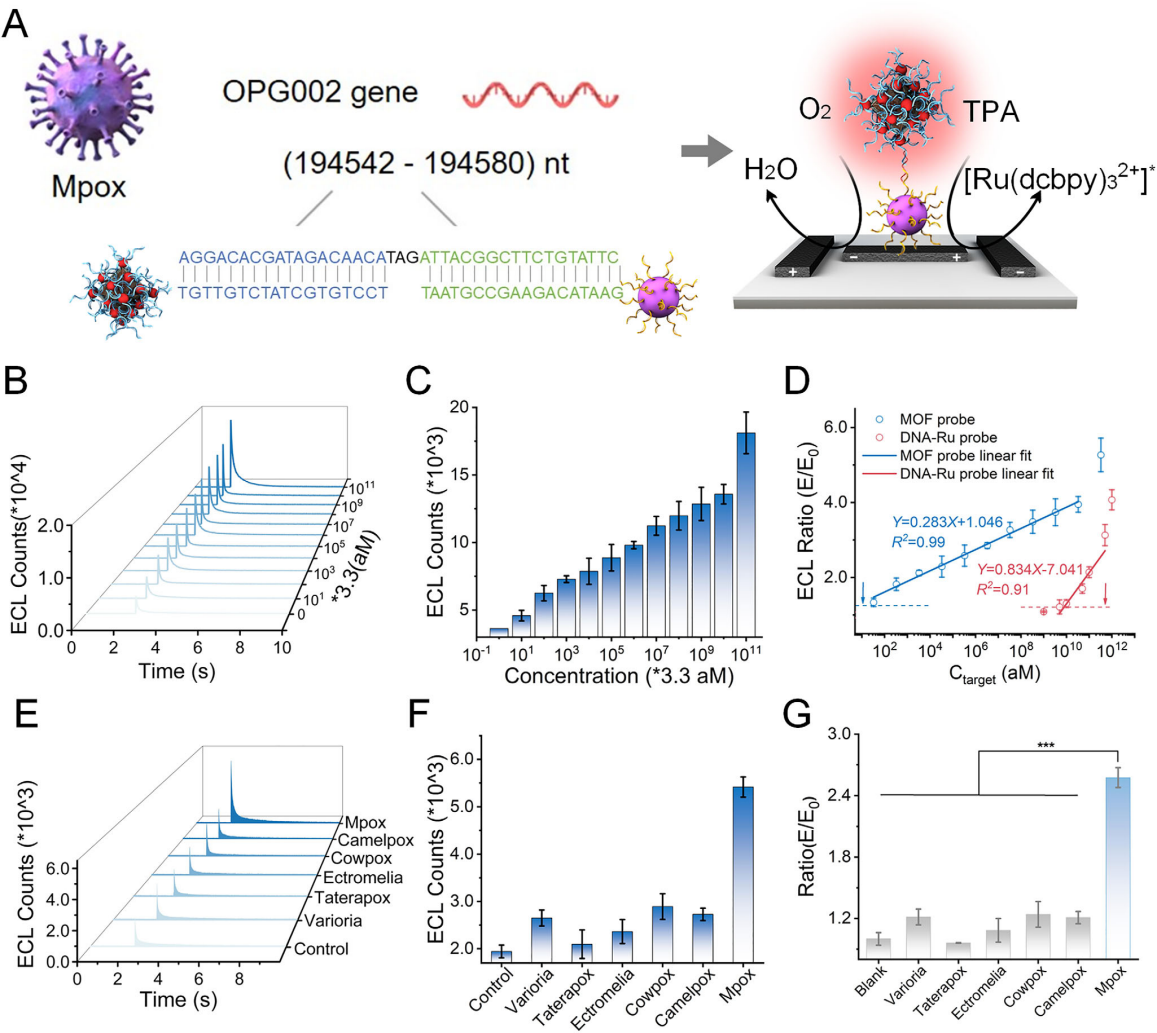
Establishment of cage‐type three‐dimensional (3D) electrochemiluminescence (ECL) probe‐enhanced nucleic acid assay. (A) Schematic of cage‐type 3D ECL probe‐enhanced hybridization assay for monkeypox (Mpox). (B,C) ECL profiles of the sensor toward different concentrations of Mpox target. (D) ECL ratio (*E*/*E*_0_)‐based calibration plot of the ECL sensor for the Mpox target. (E,F) ECL profiles of the sensor toward Mpox target with different homologous interferents. (G) ECL ratio profiles of the sensor for Mpox target (10 pM) with different homologous interferents (each at 100 pM). A *p*‐value of < 0.05 was considered statistically significant (*p < 0.05; **p < 0.01; ***p < 0.001).

The sensitivity and specificity are key factors for the feasibility of the cage‐type 3D ECL probe‐enhanced nucleic acid assay. Herein, a specific probe sequence was designed for targeting the Mpox‐secreted tumor necrosis factor‐alpha‐receptor‐like protein, and the synthesized Mpox DNA homologous fragment was used as input for validating the performance of the assay (Figure 5A). The ECL signal was collected, and the relative ECL between *E*_target_ and *E*_0_ was calculated for robust analysis of LOD corresponding to different dilutions of the synthesized Mpox DNA (Figure 5B–D). The intensity of the ECL signal gradually increased with increasing input target concentration (33–3.3 × 10^10^ aM), yielding a good linear relationship (*Y* = 0.283*X* + 1.046, *R* ^2^> 0.99) and an LOD of 5 aM (3 copies/µL). Notably, the lower LOD and broader detection range compared with those of PCR‐based approaches indicated an excellent nucleic acid detection performance [44]. In contrast, the linear range of the single Ru(dcbpy)_3_Cl_2_‐mopdified DNA probe‐based assay was 10^10^–10^12^ aM with an LOD of 9.3 nM. Hence, the LOD of the cage‐type 3D ECL probe was approximately 10^8^‐fold lower than that of the DNA‐Ru‐based assay, resulting in a highly sensitive detection of the Mpox DNA. To evaluate the selectivity of the assay, the specificity was examined using homologous sequences of Mpox, including camelpox, cowpox virus, ectromelia, taterapox virus, and variola. Notably, a significant ECL signal was achieved only in the presence of Mpox, along with low cross‐activity against other viral nucleic acids (Figure 5E–G). Overall, the cage‐type 3D ECL probe‐enhanced assay exhibited various promising results for POC diagnosis, including rapid assay time (≈10 min), excellent sensitivity (≈3 copies/µL), wide dynamic range (≈8 log), and acceptable specificity. The versatility of the assay was further explored for other pathogenic nucleic acids. The probe sequence was simply replaced with the corresponding target sequence of interest. Herein, SARS‐CoV‐2 (nucleocapsid phosphoprotein, N, 28840–28879) was selected as a model (Figure S7a, Supporting Information). The Fe‐MOF‐enhanced ECL hybridization assay was performed as described, and the sequences of capture and reporter probes were tailored for SARS‐CoV‐2 RNA. Notably, the ECL assay showed comparable performance in detecting SARS‐CoV‐2 to that of Mpox, achieving an LOD of 5 aM and a linear detection range of 33–3.3 × 10^10^ aM (Figure S7b,c, Supporting Information). Furthermore, the specificity of the assay was examined using homologous sequences of SARS‐CoV‐2 (Figure S7d, Supporting Information), and only the presence of SARS‐CoV‐2 target yielded a significant signal, along with low cross‐activity against other viral nucleic acids.

### 2.6 Validation of POC Detection of Mpox and SARS‐CoV‐2 Using Clinical Samples

**FIGURE 6:**
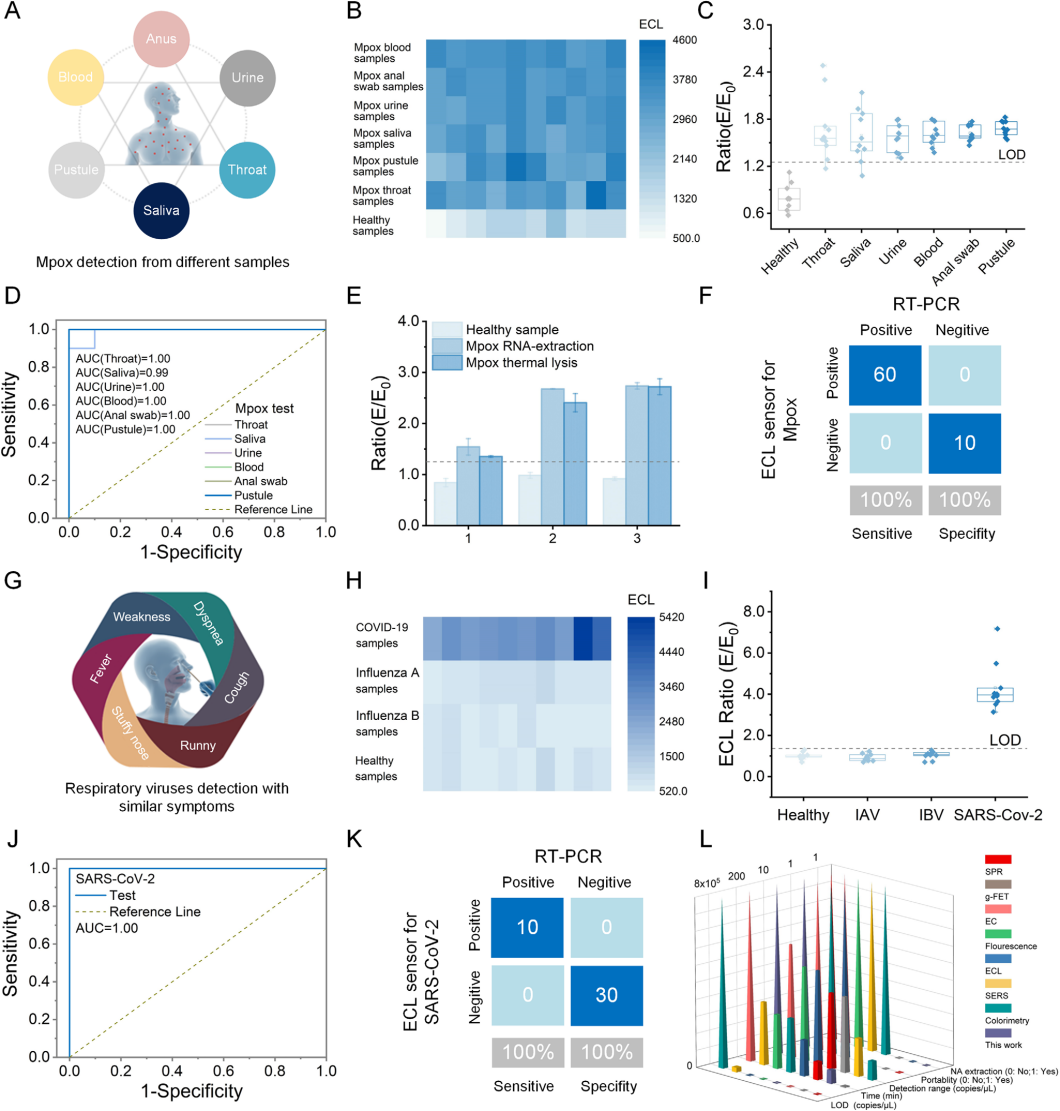
Clinical validation of cage‐type three‐dimensional (3D) electrochemiluminescence (ECL) probe‐enhanced nucleic acid assay. (A) Schematic diagram for the sources of monkeypox (Mpox) clinical samples collected from various body fluids. (B,C) ECL signal and ECL ratio (*E*/*E*_0_) response in different Mpox samples. (D) Receiver operating characteristic (ROC) curves of the ECL assay for detecting RNA target in different samples of 10 patients with Mpox and 10 healthy donors. (E) Analytical performance of thermal lysis and RNA extraction of Mpox clinical samples. (F) Evaluation of ECL sensor for Mpox sensitivity and specificity compared to standard reverse transcription polymerase chain reaction (RT‐PCR) test using a confusion matrix. (G) Schematic diagram for the sources of severe acute respiratory syndrome coronavirus 2 (SARS‐CoV‐2) clinical samples collected from nasopharyngeal swabs. (H,I) ECL signal and ECL ratio (*E/E*_0_) response in nasopharyngeal swab samples of patients with coronavirus disease 2019 and healthy donors. (J) ROC curves of the ECL assay for detecting RNA target of SARS‐CoV‐2 in 20 human samples. (K) Evaluation of the ECL sensor for SARS‐CoV‐2 sensitivity and specificity compared with those of the standard RT‐PCR test using a confusion matrix. (L) The current state of representative amplification‐free and ultrasensitive nucleic acid biosensing approaches.

Owing to the excellent performance of the cage‐type 3D ECL probe‐enhanced nucleic acid assay, its potential applicability for clinical samples was evaluated (Figure 6A). First, its performance for Mpox samples was analyzed. Although Mpox is a double‐stranded DNA virus, Mpox RNA transcripts (OPG002 gene, 194554–194592) from infected individuals have been confirmed [45]. Because Mpox shedding can occur across multiple body sites, various body fluid samples, including pustule, blood, urine, anal swab, saliva, and throat swabs, were collected from 10 patients with Mpox and 10 healthy volunteers, with each sample being validated by qPCR (Figures S8–S14, Supporting Information). The proposed assay correctly identified all Mpox‐positive and ‐negative samples, with markedly enhanced ECL signal and ECL ratio (*E*/*E*_0_) in all positive samples (Figure 6B,C). Interestingly, the sample collected from the saliva, blood, urine, and anal swabs showed a smaller deviation than those obtained from throat swabs and pustule samples. This may be attributed to sampling bias. Nevertheless, the proposed assay could effectively detect Mpox regardless of the sampling sites. Furthermore, the optimal cutoff value of ECL ratio for Mpox was determined to be 1.25 for the best predictive value, yielding an area under the curve (AUC) of > 0.99 (*p* < 0.001) for each sample type (Figure 6D). To simplify the entire workflow, the effect of the sample preparation method on our assay was assessed by thermal lysis and using an RNA extraction kit. Notably, both sample preparation methods led to a similar ECL ratio in the detection of each sample (Figure 6E). This indicated the robustness of the assay, allowing for a simple sample preparation procedure such as thermal lysis. Additionally, the assay exhibited 100% sensitivity and specificity when validated against RT‐qPCR results for the same clinical samples (Figure 6F).

The efficacy of the assay in the successful detection of Mpox was further validated through COVID‐19 diagnosis. Herein, the assay was performed to analyze clinical nasopharyngeal swab RNA extracts (Figure 6G) collected from 10 suspected patients with COVID‐19 and 10 healthy volunteers, which were validated by RT‐qPCR (Figure S15, Supporting Information). The proposed assay correctly identified all COVID‐19‐positive and ‐negative samples, resulting in significantly enhanced ECL signal and ECL ratio (*E*/*E*_0_) in all positive samples (Figure 6H,I). Moreover, the optimal cutoff value of ECL ratio was determined to be 1.36 for the best predictive value, yielding an AUC of 1.0 (*p* < 0.001), with 100% sensitivity and specificity (Figure 6J,K). Overall, these results show that the assay proposed in this study can provide rapid and accurate detection of pathogenic nucleic acids even with unextracted samples, with the aim of enabling the key steps for a POC diagnostic.

### 2.7 Comparative Performance Analysis of Amplification‐Free and Ultrasensitive Nucleic Acid Biosensors

The development of amplification‐free and ultrasensitive nucleic acid biosensors is critical for rapid and accurate pathogen detection [46]. Presently, amplification‐free nucleic acid detection methods mainly utilize CRISPR [47, 48, 49, 50, 51, 52, 53, 54], SPR [55, 56], EC [57], ECL [58, 59], and their combinations [60, 61]. To highlight the advantages of the cage‐type 3D ECL probe‐enhanced nucleic acid assay proposed in this study, its performance was systematically compared with that of existing nucleic acid detection methods (Figure 6L and Table 1). As a representative biochemical signal amplification strategy, CRISPR/Cas systems possess a target‐dependent trans‐cleavage mechanism, which results in highly specific target recognition and highly processive cleavage of nearby reporter molecules. Ultimately, they exhibit desirable signal transduction capability and versatile applicability in the combination of diverse signal readouts, including SERS [46, 49], colorimetric [51], fluorescent [52], g‐FET [53], EC [57], and ECL [58]. Furthermore, physical mechanisms for signal‐amplification, such as confinement effect [48] and SPR [8], greatly contribute to sensitivity improvement. Both EC and ECL present considerable advantages, such as low cost, simple structure, miniaturization, and high sensitivity. However, complex clinical signals can interfere with the readings of EC sensors, affecting the accurate analysis of unpurified clinical samples, especially in samples with low‐abundance targets. In contrast, ECL generally has a high *S*/*N*, due to its unique luminescent mechanism [62]. The existing ECL methods for nucleic acid testing mainly focus on improving the luminescent efficiency of ECL probes to further improve analysis performance. However, these efforts have been limited by various factors, including detection sensitivity, detection time, and portability. Compared with these methods, the presented cage‐type 3D ECL probe‐enhanced nucleic acid assay exhibited the advantages of higher sensitivity, shorter turnaround time, broader dynamic range, and portability. Additionally, the proposed assay was compatible with thermally lysed clinical samples without the need for further nucleic acid extraction. Owing to its simplicity, a portable ECL detection instrument coupled with a low‐cost p‐BPE ECL biosensor was constructed for rapid, portable, and ultrasensitive POC detection of Mpox and SARS‐CoV‐2. Altogether, the results showed that the proposed assay has the potential to be a promising paradigm for POC diagnosis of infectious diseases.

**TABLE 1:** Performance of amplification‐free and ultrasensitive nucleic acid biosensors.

Assay method	Test sample	Detection range (copies/µL)	LOD (copies/µL)	Portability	Nucleic acid extraction	Time [min]	Reference
Cascaded CRISPR	RNA	10^4^–10^5^	2.1 × 10^3^	×	√	100	[47]
Ultralocalized CRISPR	RNA	10^1^–10^5^	6	×	√	60	[48]
CRISPR + SERS	DNA	10^4^–10^5^	2.2 × 10^4^	×	√	70	[49]
CRISPR +ICP‐MS	DNA	10^4^–10^6^	3.3 × 10^4^	×	√	110	[50]
CRISPR + colorimetry	RNA	/	8.0 × 10^5^	√	√	60	[51]
CRISPR + fluorescence	RNA	/	100	√	√	30	[52]
Y‑shaped probe + g‐FET	RNA	10^2^–10^5^	0.03	×	×	1	[28]
CRISPR/Cas9 + g‐FET	DNA	10^3^–10^4^	3.8 × 10^3^	√	√	15	[53]
Aromatic ring‐mediated g‐FET	RNA	0.6−10^3^	3	×	√	120	[62]
CRISPR/Cas12a+g‐FET	DNA	10^−2^–10^12^	5	×	×	20	[63]
CRISPR‐SPR‐FT sensor	DNA	10^2^–10^6^	59.5	√	√	90	[8]
Graphdiyne‐based SPR	DNA	10^2^–10^5^	800	×	√	50	[65]
Quantum dot‐based SPR	RNA	10^−3^–1	0.008	×	√	20	[66]
RCA + EC	RNA	1–10^9^	1.0	√	√	120	[57]
EC biosensor Chip	RNA	10^2^−10^7^	6.9	√	√	5	[31]
Smartphone + EC	RNA	16−10^6^	0.2	√	√	190	[32]
CRISPR + ECL	DNA	10^6^–10^10^	7.7 × 10^5^	×	√	80	[58]
Interposed [Ru(phen)_2_dppz]^2+ ^+ ECL	DNA	10^1^–10^4^	0.05	×	√	120	[35]
Linear Ru(bpy)_3_^2+^‐Polymer + ECL	DNA	10^1^–10^5^	60	×	√	30	[38]
Dendritic Ru(bpy)_3_^2+^‐Polymer+	RNA	10^3^–10^7^	500	×	√	40	[37]
ECL Ru‐MOF	RNA	10^2^–10^6^	180	×	√	240	[59]
Ru(dcbpy)_3_^2+^ doped MOF+ECL	RNA	20–10^10^	3.3	√	×	10	This work

## 3 Conclusion

In this study, a cage‐type 3D ECL reporter probe was fabricated for amplification‐free and ultrasensitive detection of pathogenic nucleic acids. The high loading capacity of luminescent molecules, along with PDA‐ and SNA‐mediated surface modifications, resulted in a significant enhancement (10^3^‐fold) in ECL performance and chemical stability (with minimal leakage of Ru(dcbpy)_3_Cl_2_). Additionally, the findings of this study showed that the enhanced ECL assay exhibited excellent performance in terms of assay time (15 min), LOD (3 copies/µL), broad detection range (20–10^10^ copies/µL), and simple operation. Moreover, a portable ECL detection instrument coupled with a low‐cost pBPE ECL biosensor was constructed in this study, allowing for rapid, portable, and ultrasensitive POC detection of Mpox and SARS‐CoV‐2. Altogether, the findings of this study show the potential of the developed SNA‐stabilized cage‐type 3D ECL probe‐enhanced nucleic acid assay for POC diagnosis of infectious diseases.

## 4 Experimental Section

### 4.1 Synthesis of the Cage‐Type 3D ECL Probe (Fe‐Ru‐MOF‐PDA‐SNA)

The cage‐type 3D ECL probe, namely Ru(dcbpy)3Cl_2_‐doped MIL‐101(Fe)(Fe‐Ru‐MOF), was synthesized as described previously, with some modifications [63]. FeCl_3_·6H_2_O (0.11 g, 0.4 mmol), NH_2_‐BDC (0.0375 g, 0.2 mmol), and Ru(dcbpy)_3_Cl_2_ (1.8 mg, 2.0 µmol) were dissolved in 30 mL of *N*,*N*‐dimethylformamide and subjected to ultrasound for 10 min. Next, the mixture was transferred to a Teflon‐lined stainless‐steel bomb and heated at 110°C for 20 h. Following this, 10 mg of Fe‐Ru‐MOF and 15 mg of DA were dispersed in 100 mL of a mixed solution (EtOH:H_2_O = 2:1) [64]. The mixture was transferred to a 100‐mL round‐bottom flask and heated at 60°C for 2 h. Afterward, MOF‐PDA (1 mL, 1 mg mL^−1^) was mixed with SNAs (1 mL, 10 nM) at room temperature for 1 h, followed by the addition of NaCl (30 mM) and mixing for another 1 h. To block non‐specific sites on the Fe‐Ru‐MOF‐PDA‐SNA, 10 µM DNA sequence homologous to the capture probe was added, followed by a 12‐h incubation. Finally, the sediment was obtained after centrifugation (5000 rpm), and after washing thrice with ultrapure water, the resulting Fe‐Ru‐MOF‐PDA‐SNA was stored at room temperature until further use.

### 4.2 Operational Protocol

Unless otherwise indicated, 5 µL of sample lysate or purified DNA sequence homologous to the RNA target was added to the 500 µL of reaction solution, containing 0.4 mg·mL^−1^ MB‐CP (either for Mpox or SARS‐Cov2), 50 µL of Fe‐MOF reporter probe (1 mg·mL^−1^), and 50 µL of NaCl (1 M). The mixture was then incubated at 37°C for 10 min, and the complex was isolated by magnetic separation, followed by three times washing with ultrapure water (1 mL each). Afterward, the resulting sediment obtained after centrifugation was mixed with 15 µL of TPA auxiliary solution and deposited onto the bipolar electrode of the paper‐based ECL sensor chip. The chip was inserted in the portable detection device, and the ECL signal was obtained at 20 V working voltage and 10 s integral time.

### 4.3 Clinical Sample Pre‐Treatment

Herein, the clinical samples from the urine and plasma were directly used for nucleic acid extraction. In contrast, the swabs from the throat, pustule, and rectum, along with saliva samples, were first dissolved with 2 mL of viral transport medium [65]. Next, the nucleic acid was extracted using a commercial instrument (Smart 32 Plus, DaAn Gene Co., Ltd, Guangzhou).

## Author Contributions

Yu Fu, Wenlu Song, Bowen Shu, and Yuhui Liao conceived the idea and designed experiments. Jinyan Lin, Wei Wang, Yuhui Liao, Yideng Jiang, and Yang Yang collected the clinical samples. Yu Fu, Wenlu Song, Huiyi Yang, and Lunjing Liu designed and synthesized the self‐enhanced ECL probe. Yu Fu and Wenjie Wu built the pBPE and portable detection device. Wenlu Song, Jinyan Lin, Jingru Wang, Lunjing Liu, and Wei Wang analyzed and verified the data. Yuhui Liao, Bowen Shu, Yideng Jiang, and Yu Fu contributed to funding acquisition. Wenlu Song, Yu Fu, and Bowen Shu edited the manuscript. Yideng Jiang, Yang Yang, Bowen Shu, and Yuhui Liao reviewed the experimental data and manuscript. All authors have given approval to the final version of the manuscript.

## Conflicts of Interest

The authors declare no conflicts of interest.

## Ethics Statement

The studies involving nucleic acid samples from human subjects were reviewed and approved by the Shenzhen Third People's Hospital (No.2020‐0127; No. 2021–030). All subjects provided written informed consent.

## Supporting information




**Supporting file 1**: exp270154‐sup‐0001‐SuppMat.pdf.

## Data Availability

The data that support the findings of this study are available from the corresponding author upon reasonable request.
