# Supplementary material for: Spherical Nucleic Acid Stabilized Cage Type Three‐Dimensional Electrochemiluminescence Probe for Sensitive and Amplification‐Free Diagnosis of Infectious Diseases
Source: Exploration (Beijing). 2026 Mar 14;6(2):20240451. doi: 10.1002/EXP.20240451 (PMC13094524; doi:10.1002/EXP.20240451)
Supplement: Supplementary file 1 — Supporting file 1: exp270154‐sup‐0001‐SuppMat.pdf. [file EXP2-6-20240451-s001.pdf]

Supporting Information

Spherical Nucleic Acid Stabilized Cage Type Three-dimensional Electrochemiluminescence Probe for Sensitive and Amplification-free Diagnosis of Infectious Diseases

1. Experimental section

1.1 Materials

Analytical-grade reagents were used in this study. Iron chloride hexahydrate ($FeCl_3 \cdot 6H_2O$), 2-aminoterephthalic acid (NH_2-BDC), tri-(4,4-dicarboxybipyridine), ruthenium chloride ($Ru(dcbpy)_3Cl_2$), and sodium chloride were purchased from Aladdin (Shanghai, China). *N,N*-dimethylformamide, anhydrous ethanol, and dopamine were purchased from Macklin (Shanghai, China). Chloroauric acid was obtained from ACMEC (Shanghai, China). Streptavidin Magnetic Beads (MBs) and RNase inhibitors were purchased from MCE (New Jersey, USA). Influenza virus standards were purchased from the National Institute for Food and Drug Control (Guangzhou, China). The RNA extraction kit was purchased from TIANGEN (Beijing, China). Tripropylamine auxiliary solution was purchased from Roche (Switzerland), and all synthetic oligonucleotides (Table S1) were purchased from Shanghai Sangon Biological Engineering Technology (Shanghai, China).

1.2 Preparation of spherical nucleic acid (SNA)

AuNPs (13 nm) were prepared by citrate reduction of $HAuCl_4 \cdot 4H_2O$ based on a classic method[1]. The SNA were synthesized according to a previous study, with certain modifications[2]. Thiolated DNAs (COVID-N-PS or MPXV-PS) were treated with TCEP (DNA:TCEP=1:10) at pH 5.0 for 1 h to cleave the disulfide bond. These activated DNAs (100 µM, 10 µL) were mixed with AuNPs (10 nM, 1 mL), and then, a small volume of 200 mM citrate-HCl buffer (pH3.0) was rapidly added to the DNA

and AuNP mixture followed by pipette mixing for 15 min. The final buffer concentration was 10 mM. A small volume of 500 mM HEPES buffer (pH 7.6) was rapidly added to the mixture for 15 min, and the final buffer concentration was 20 mM. The sediment was isolated by centrifugation (8000 rpm) and washed three times with ultrapure water.

1.3 Synthesis of magnetic beads(MB) modified by capture probe(CP)

In accordance with the instructions provided by the manufacturer of the streptavidin magnetic beads, the beads were fully suspended, 100 μL of magnetic beads were transferred to a fresh 1.5 mL EP tube, and placed on a magnetic rack for separation, and the supernatant was discarded. Then, 1 mL of Tris-HCl buffer (pH 7.5, 1 mM EDTA, 1 M NaCl, 0.01–0.1% Tween-20) was added to thoroughly wash the magnetic beads, magnetically separate them, and the supernatant was discarded. Biotinylated nucleic acid (500 μL, final concentration = 3 μM), was shaken and suspended thoroughly, and incubated on a rotary mixer (room temperature, 30 min) to construct the MB-DNA. The MB-DNA were then separated, and washed with three times. Finally, 500 μL of ultrapure water was added and the beads were resuspended.

1.4 Calculation formula for Fe-Ru-MOF

$$N = \frac{\rho V}{1-P}\frac{P}{Mr}N_A$$

where ρ=1.1374 g/cm^3 is the density of the MOF[3], V is the volume of the MOF, P is the mass percentage of $Ru(dcbpy)_3Cl_2$, Mr is relative molecular mass of $Ru(dcbpy)_3Cl_2$, N_A =6.02×10^{23} mol^{-1} is Avogadro's constant.

2. Tables

Table S1 Sequence of the oligonucleotides used in this study.

Name	Sequences (5' to 3')
COVID-N-TS	TGCTCTTGCTTTGCTGCTGCTTGACAGATTGAACCAGCTT
COVID-N-CS	Bio-AAAAAAAAAAAAG CTG GTT CAA TCT GTC
COVID-N-PS	AGCAGCAAAGCAAGAGCAAAAAAAAAAA
H1N1-TS	CTGGCCACAGGATTGAGGAATATCCCGTCTATTCAATCT
H3N2-TS	AATGTACCAGAGAAACAAACTAGAGGCATATTTGGCGCA
BV-TS	CCAAACTGTTTTAACTGCACAGATCTGGACGTGGCCCTA
MPXV-TS	AGGACACGATAGACAACATAGATTACGGCTTCTGTATTC
MPXV-CS	Bio-AAAAAAAAAAGAATACAGAAGCCGTAAT
MPXV-PS	TGTTGTCTATCGTGTCCTAAAAAAAAAA-SH
Monkeypox	TTTACATTTTCCATTGGATGGTGCATGTGGTGCTAAATC
Camelpox	TTTACACTTTCCATTGGATGGTGCATACGGTGTTACATC
Cowpox	TTTACACTT TCCGTTGGATGGTTCATGCGG TGTTATATC
Ectromelia	GTTACATTTCCCATTAATGGGTGTATACGGAACATCTCC
Taterapox	TTTACACTTTCCATTGGATGGTGCATACGGTGCTACATC
Varioria	ATTACTGTTCCCATGGCTATTACCCACTGTCCATCCACA
Mpox forward primer	ACGCTAAATTAACGTCTACCGAAACATCGT
Mpox reverse primer	GGTGGAATTCACTTCGTATAATGGCTTATC

Table S2 Detail information of clinical tissue samples.

Patient ID	Sex	Age	Diagnosis
1	M	22	COVID-19
2	M	15	COVID-19
3	M	30	COVID-19
4	F	26	COVID-19
5	F	23	COVID-19
6	M	48	COVID-19
7	M	21	COVID-19
8	F	32	COVID-19
9	F	36	COVID-19
10	M	45	COVID-19
11	M	29	Mpox
12	M	29	Mpox
13	M	21	Mpox
14	M	32	Mpox
15	M	30	Mpox
16	M	23	Mpox
17	M	28	Mpox
18	M	27	Mpox
19	M	21	Mpox
20	M	37	Mpox
21	M	42	Mpox
22	M	24	Mpox
23	M	28	Mpox

Table S3 Ct values measured by RT-qPCR for COVID-19 samples.

Clinical samples	Ct vaule	Clinical samples	Ct vaule
healthy volunteer 1	NA	COVID-19 sample 1	24.1
healthy volunteer 2	NA	COVID-19 sample 2	26.8
healthy volunteer 3	NA	COVID-19 sample 3	23.8
healthy volunteer 4	39.7	COVID-19 sample 4	20.0
healthy volunteer 5	NA	COVID-19 sample 5	19.5
healthy volunteer 6	NA	COVID-19 sample 6	21.8
healthy volunteer 7	39.9	COVID-19 sample 7	22.7
healthy volunteer 8	NA	COVID-19 sample 8	22.3
healthy volunteer 9	NA	COVID-19 sample 9	27.6
healthy volunteer 10	NA	COVID-19 sample 10	24.2

Table S4 Ct values measured by RT-qPCR for MPXV samples.

Clinical samples	Ct vaule	Clinical samples	Ct vaule	Clinical samples	Ct vaule
healthy volunteer 1	NA	MPXV herpes sample1	28.1	MPXV anal swab 1	31.0
healthy volunteer 2	NA	MPXV herpes sample2	28,4	MPXV anal swab 2	31.2
healthy volunteer 3	NA	MPXV herpes sample3	30.2	MPXV anal swab 3	31.3
healthy volunteer 4	NA	MPXV herpes sample4	29.6	MPXV anal swab 4	30.59
healthy volunteer 5	NA	MPXV herpes sample5	30.1	MPXV anal swab 5	31.3
healthy volunteer 6	NA	MPXV herpes sample6	30.7	MPXV anal swab 6	31.6
healthy volunteer 7	NA	MPXV herpes sample7	30.8	MPXV anal swab 7	30.9
healthy volunteer 8	NA	MPXV herpes sample8	31.7	MPXV anal swab 8	32.1
healthy volunteer 9	NA	MPXV herpes sample9	31.6	MPXV anal swab 9	31.7
healthy volunteer 10	NA	MPXV herpes sample10	31.6	MPXV anal swab 10	32.9
MPXV	29.3	MPXV	27.2	MPXV	33.7

throat swab 1		saliva 1		urine 1	
MPXV throat swab 2	28.7	MPXV saliva 2	27.2	MPXV urine 2	33.1
MPXV throat swab 3	28.2	MPXV saliva 3	27.9	MPXV urine 3	32.9
MPXV throat swab 4	28.4	MPXV saliva 4	28.0	MPXV urine 4	32.4
MPXV throat swab 5	30.3	MPXV saliva 5	25.5	MPXV urine 5	32.2
MPXV throat swab 6	29.9	MPXV saliva 6	25.8	MPXV urine 6	31.3
MPXV throat swab 7	30.2	MPXV saliva 7	25.9	MPXV urine 7	32.3
MPXV throat swab 8	30.1	MPXV saliva 8	25.8	MPXV urine 8	32.7
MPXV throat swab 9	31.1	MPXV saliva 9	27.0	MPXV urine 9	33.9
MPXV throat swab 10	30.7	MPXV saliva 10	28.4	MPXV urine 10	33.8
MPXV blood 1	33.3	MPXV blood 5	31.5	MPXV blood 9	32.9
MPXV blood 2	33.2	MPXV blood 6	31.3	MPXV blood 10	33.88

MPXV blood 3	33.2	MPXV blood 7	30.6
MPXV blood 4	32.8	MPXV blood 8	32.1

3. Figures

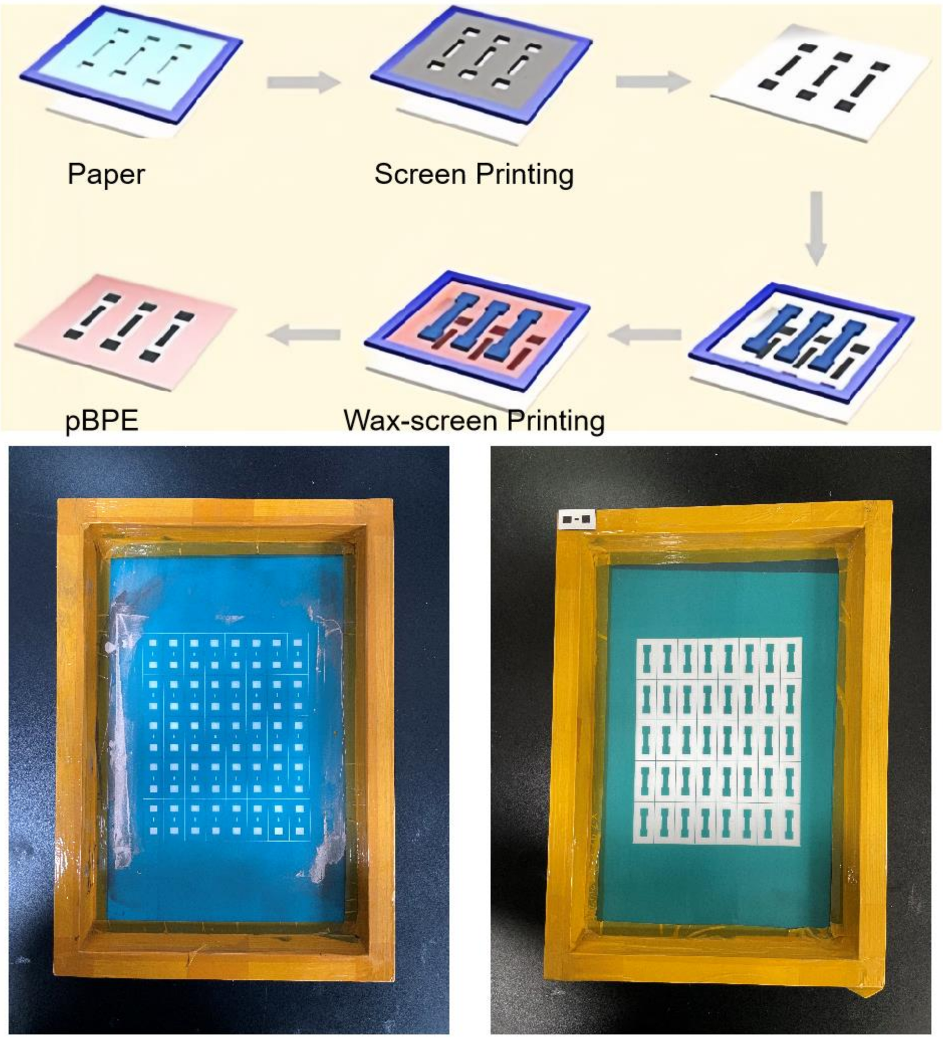

Figure S1. Production process of the detection paper chip with bipolar electrode.

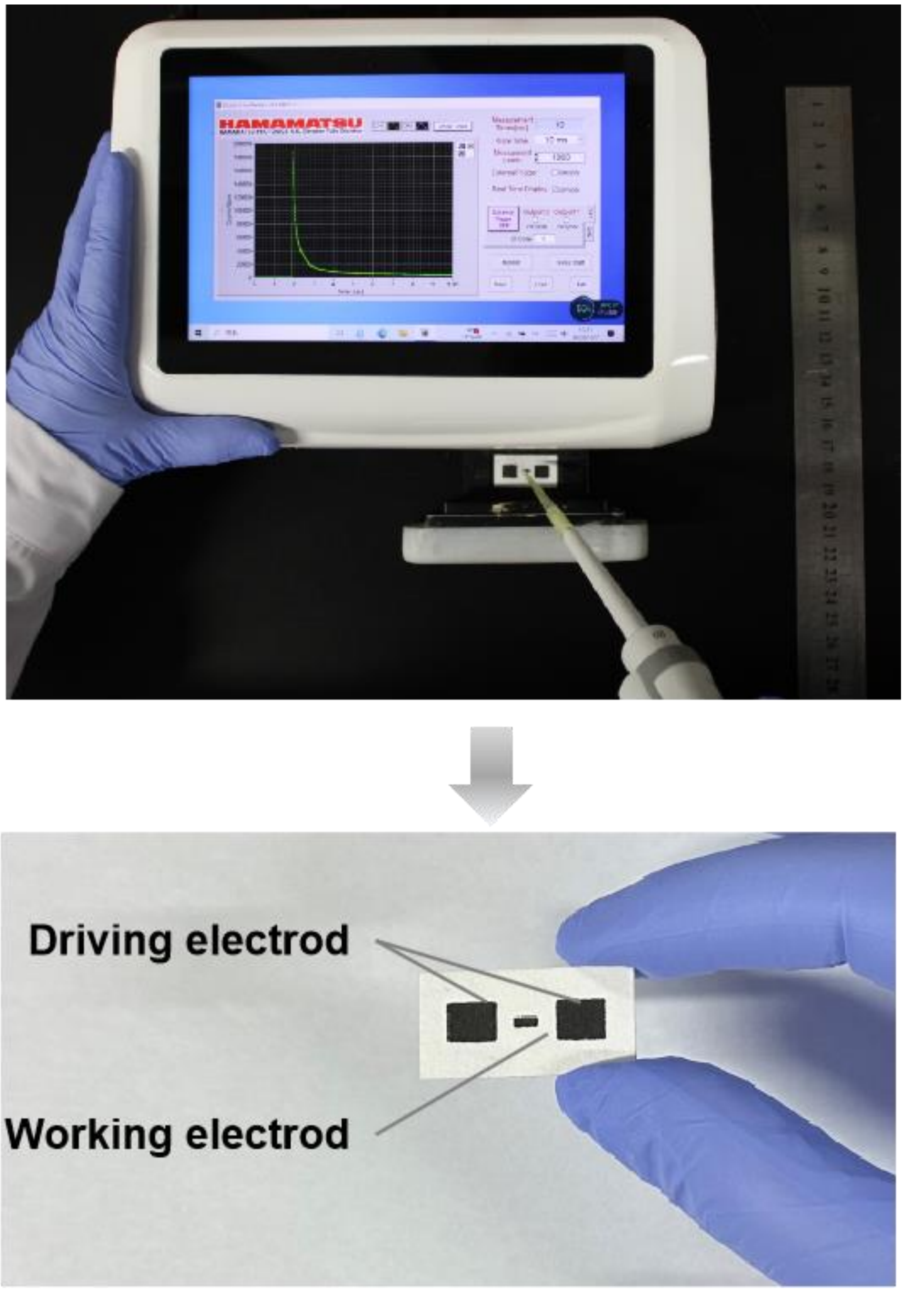

Figure S2. Image of portable detection device and detection paper chip with bipolar electrode.

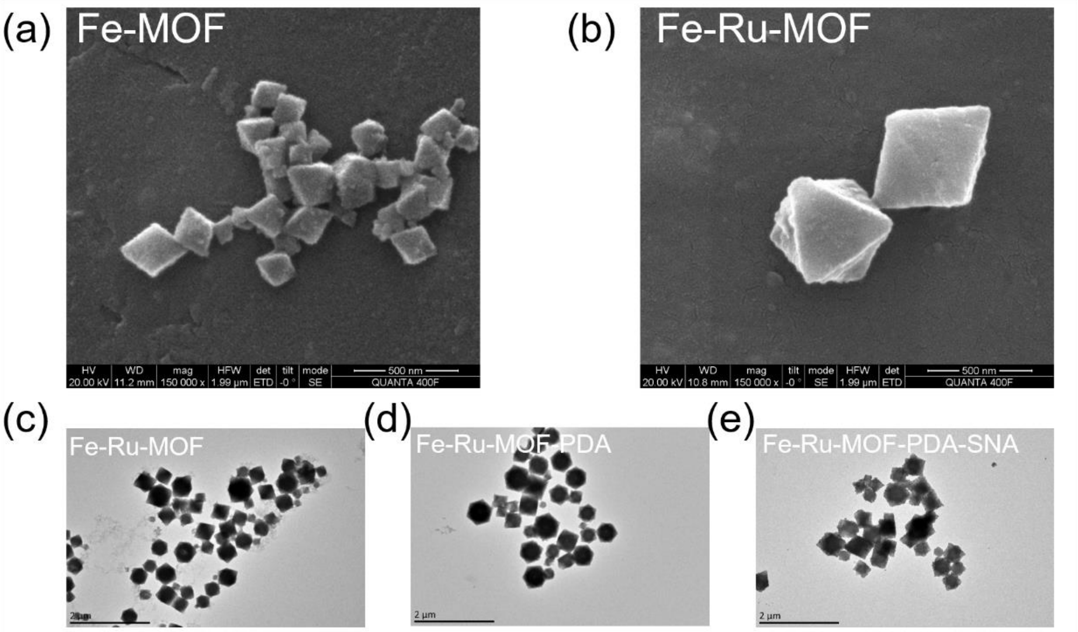

Figure S3. (a) SEM image of Fe-MOF. (b) SEM image of Fe-Ru-MOF.(c) TEM image of Fe-Ru-MOF. (d) TEM image of Fe-Ru-MOF-PDA. (e) TEM image of Fe-Ru-MOF-PDA-SNA.

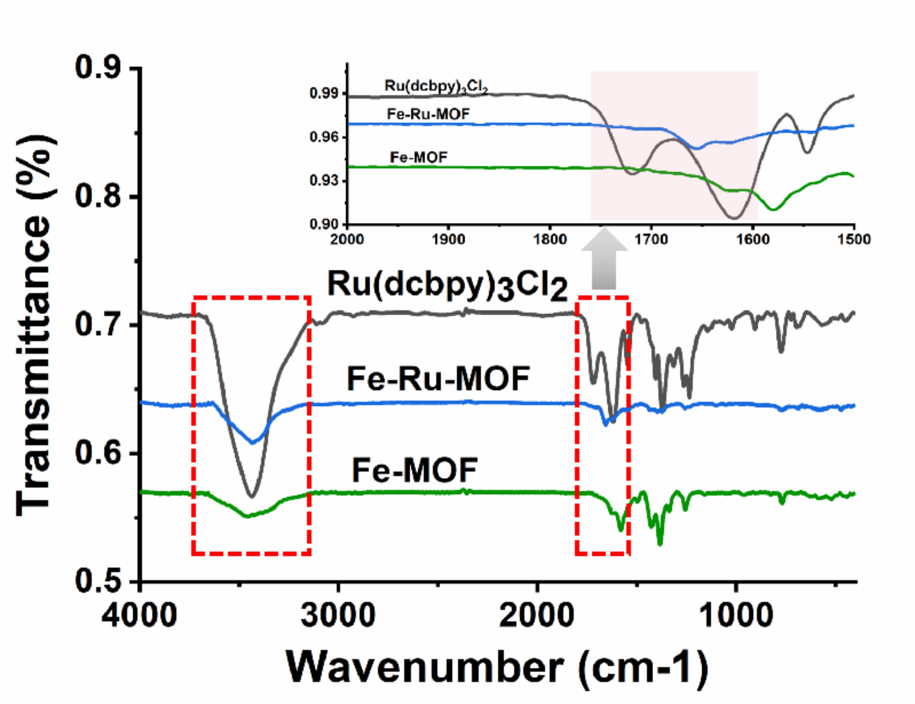

Figure S4. FT-IR spectra of Ru(dcbpy)3Cl2, Fe-MOF and Fe-Ru-MOF.

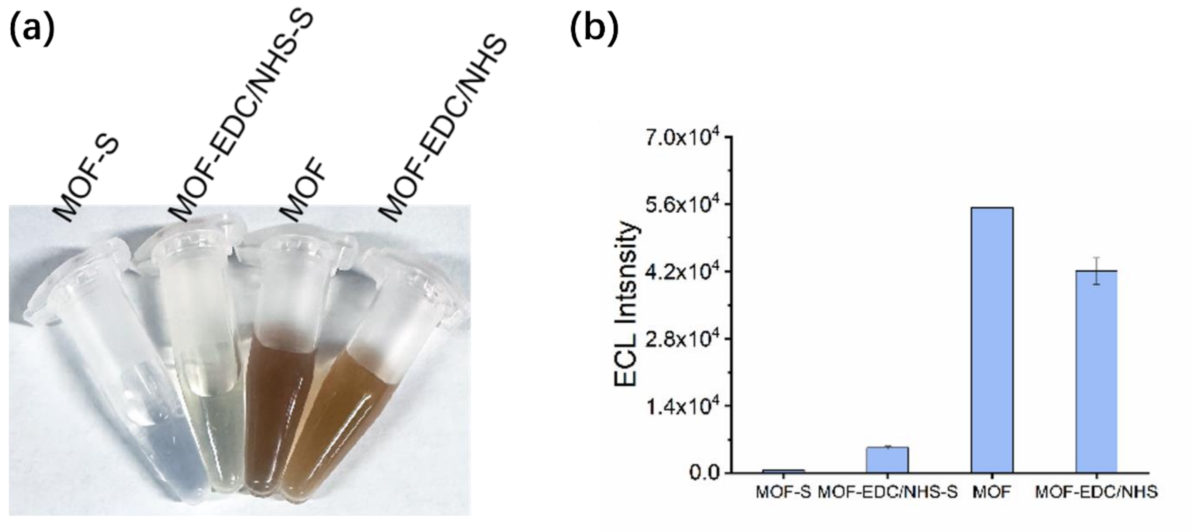

Figure S5. (a) Image of supernatant and resuspension of MOF and EDC/NHS-activated MOF. (b) ECL spectra of supernatant and resuspension of MOF and EDC/NHS-activated MOF

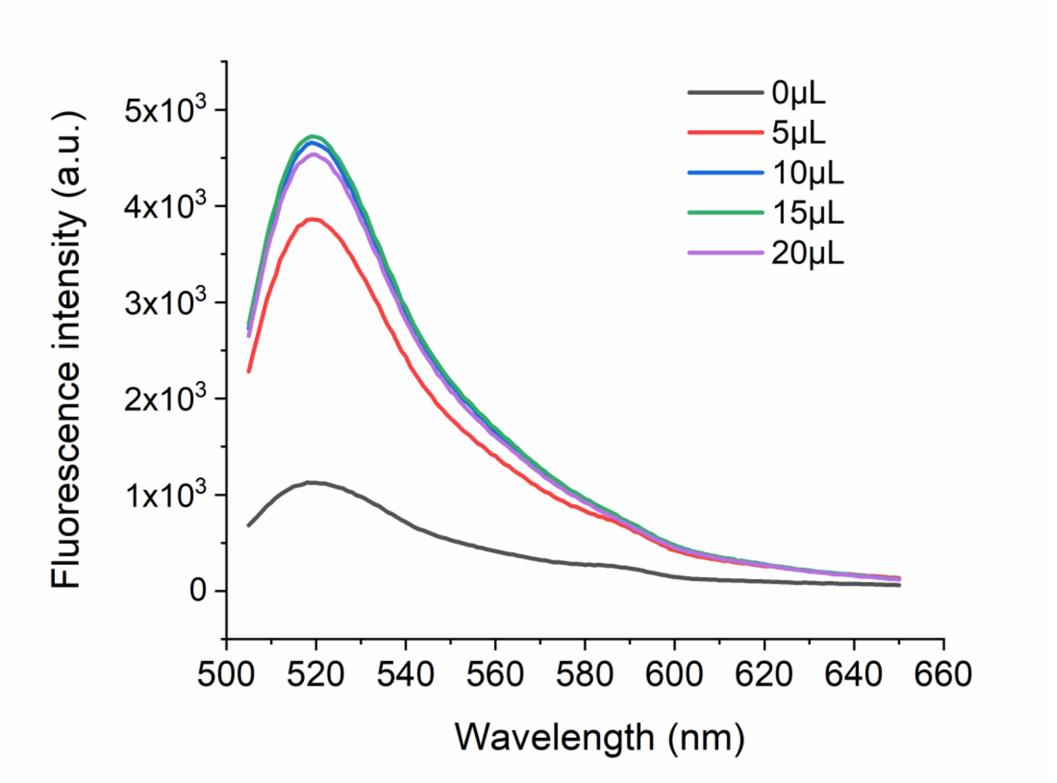

Figure S6. The test results of non-specific adsorption.

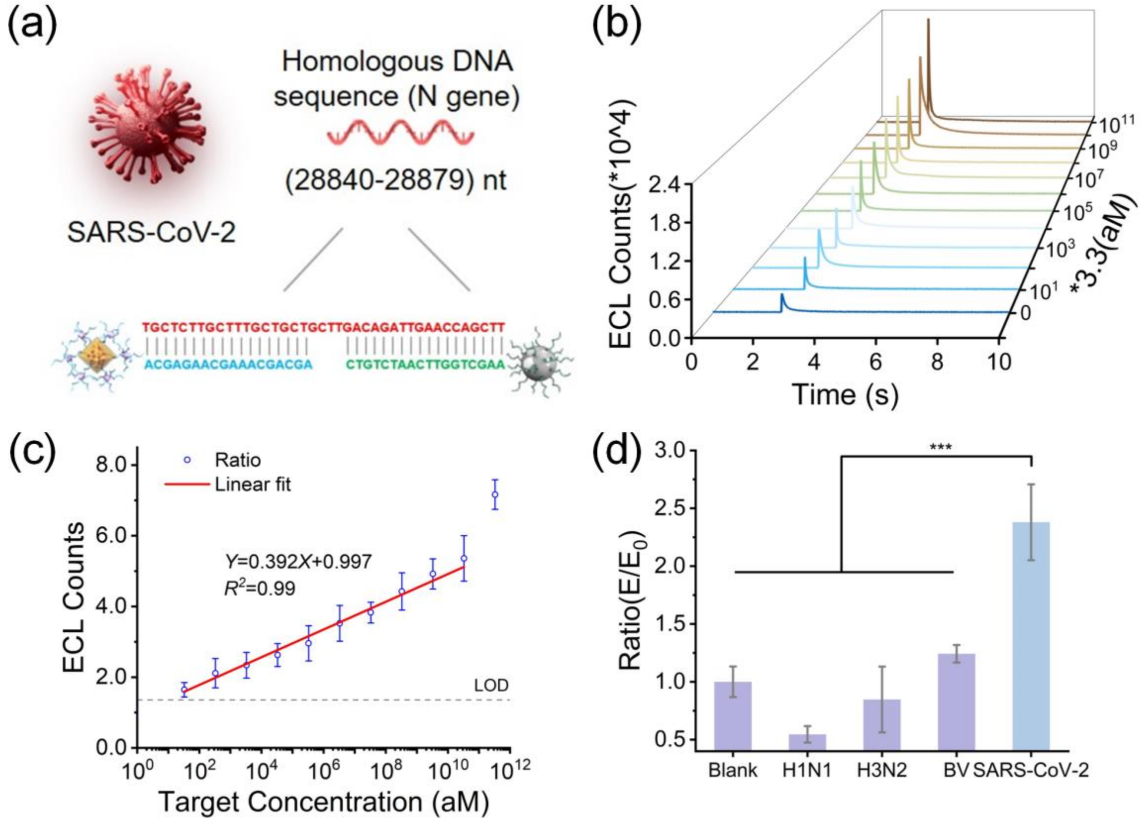

Figure S7. Versatility evaluation of cage-type 3D ECL probe enhanced nucleic acid assay. (a) Schematic of cage-type 3D ECL probe enhanced hybridization assay for SARS-CoV-2. (b) ECL profiles of the sensor toward different concentrations of SARS-CoV-2 target. (c) Calibration plot for Mpox target assay. (d) Selectivity of the ECL sensor with SARS-CoV-2 target (10 pM) and different interferents (H1N1, H3N2, BV, each at 100 pM). A P-value of <0.05 was considered statistically significant (*, $P < 0.05$; **, $P < 0.01$; ***, $P < 0.001$).

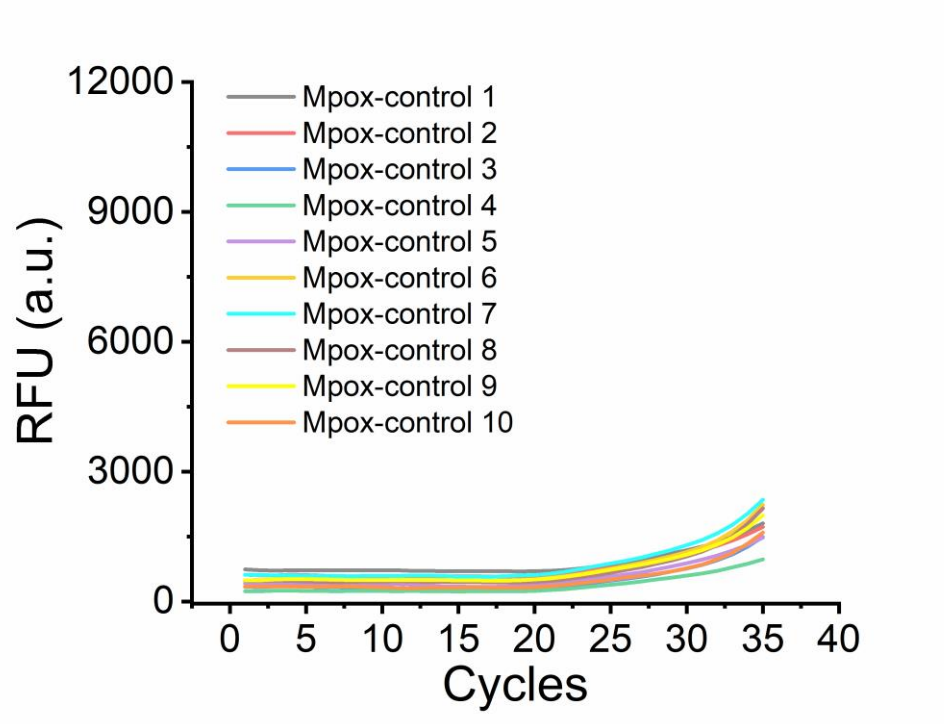

Figure S8. qRT-PCR results for Mpox detection of healthy samples.

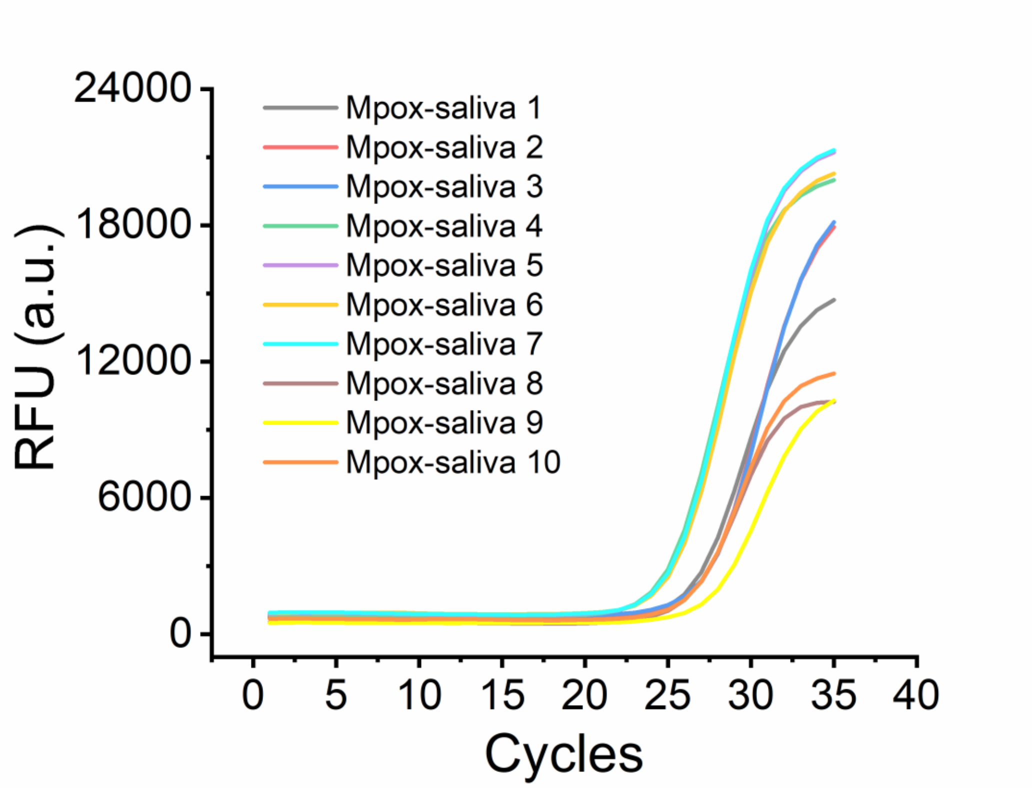

Figure S9. qRT-PCR results for Mpox detection of saliva samples.

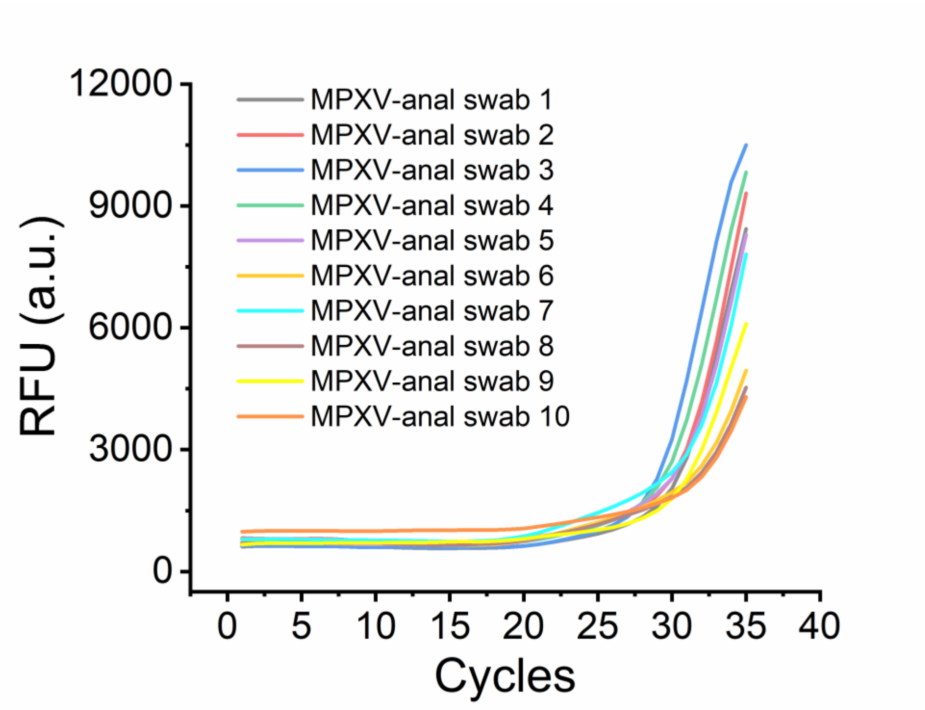

Figure S10. qRT-PCR results for Mpox detection of anal swab samples.

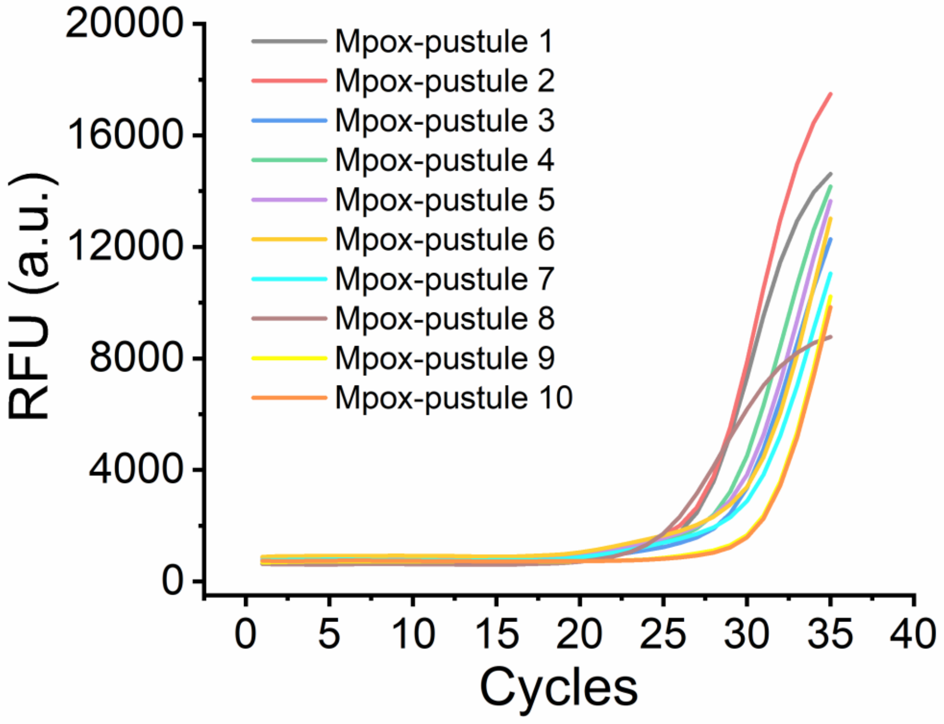

Figure S11. qRT-PCR results for Mpox detection of pustule samples.

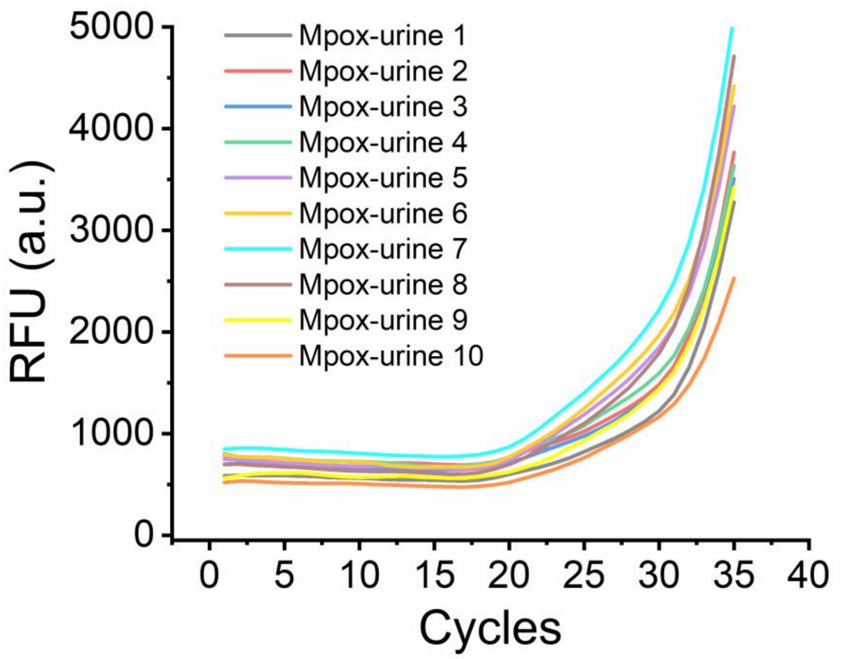

Figure S12. qRT-PCR results for Mpox detection of urine samples.

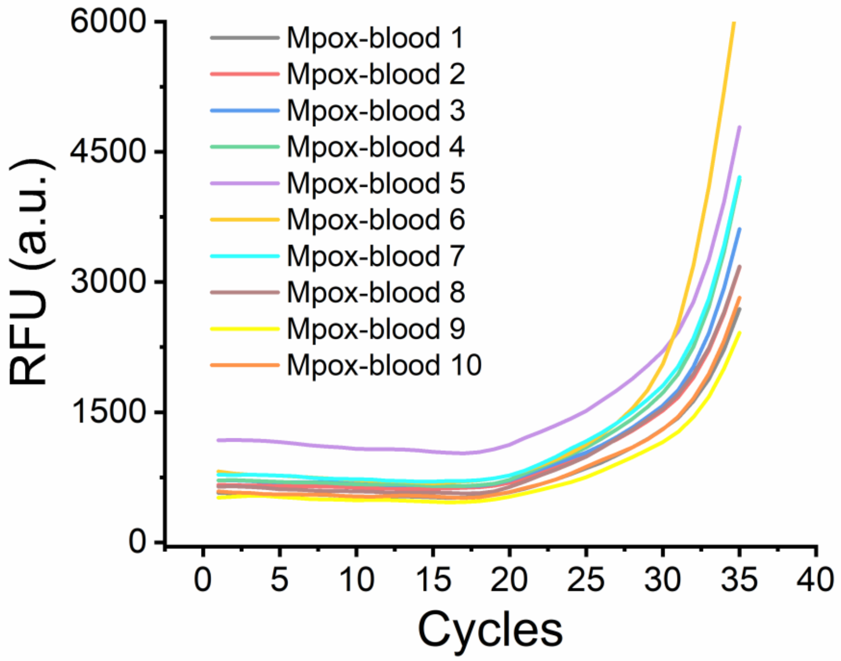

Figure S13. qRT-PCR results for Mpox detection of blood samples.

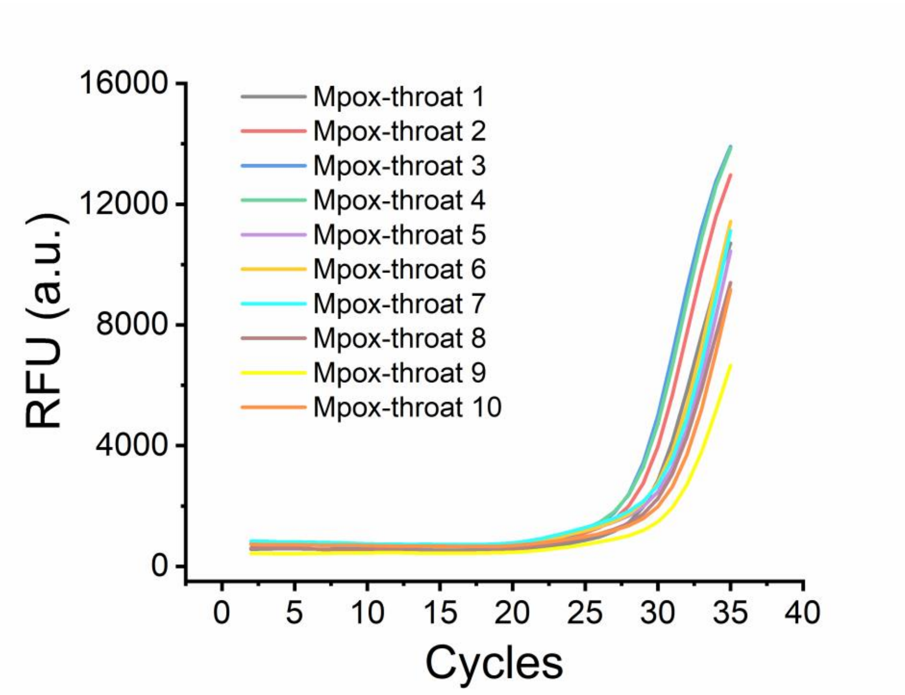

Figure S14. qRT-PCR results for Mpox detection of throat samples.

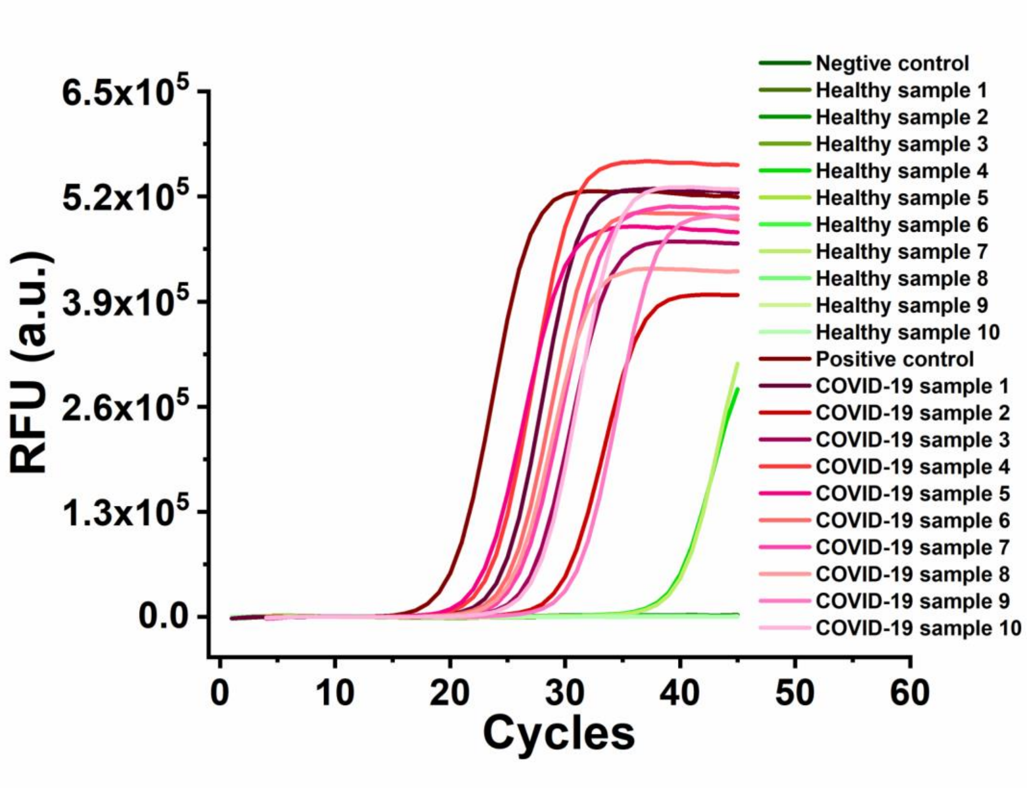

Figure S15. qRT-PCR results for COVID-19 detection.

References

[1] J. Liu, Y. Lu, *Nat Protoc* **2006**, *1*, 246-252.

[2] X. Zhang, M. R. Servos, J. Liu, *J Am Chem Soc* **2012**, *134*, 7266-7269.

[3] S. Bauer, C. Serre, T. Devic, P. Horcajada, J. Marrot, G. Férey, N. Stock, *Inorg Chem* **2008**, *47*, 7568-7576.
